# The p3 peptides (Aβ_17-40/42_) rapidly form amyloid fibrils that cross-seed with full-length Aβ

**DOI:** 10.1038/s41467-025-57341-4

**Published:** 2025-02-27

**Authors:** Yao Tian, Andrea P. Torres-Flores, Qi Shang, Hui Zhang, Anum Khursheed, Bogachan Tahirbegi, Patrick N. Pallier, John H. Viles

**Affiliations:** 1 Department of Biochemistry, School of Biological and Behavioural Sciences, Queen Mary University of London, London, E1 4NS UK; 2 The Blizard Institute, Centre for Neuroscience, Surgery and Trauma, Queen Mary University of London, London, E1 2AT UK

**Keywords:** Intrinsically disordered proteins, Prions, Protein aggregation

## Abstract

The p3 peptides, Aβ_17-40/42_, are a common alternative cleavage product of the amyloid precursor protein, and are found in diffuse amyloid deposits of Alzheimer’s and Down Syndrome brains. The p3 peptides have been mis-named ‘non-amyloidogenic’. Here we show p3_40/42_ peptides rapidly form amyloid fibrils, with kinetics dominated by secondary nucleation. Importantly, cross-seeding experiments, with full-length Aβ induces a strong nucleation between p3 and Aβ peptides. The cross-seeding interaction is highly specific, and occurs only when the C-terminal residues are matched. We have imaged membrane interactions with p3, and monitored Ca^2+^ influx and cell viability with p3 peptide. Together this data suggests the N-terminal residues influence, but are not essential for, membrane disruption. Single particle analysis of TEM images indicates p3 peptides can form ring-like annular oligomers. Patch-clamp electrophysiology, shows p3_42_ oligomers are capable of forming large ion-channels across cellular membranes. A role for p3 peptides in disease pathology should be considered as p3 peptides are cytotoxic and cross-seed Aβ fibril formation in vitro.

## Abstract (web summary)

The p3 peptide, Aβ(17-40/42), rapidly forms amyloid fibrils by secondary nucleation which cross-seeds with full-length Aβ peptide. Furthermore, p3 annular and oligomer structures are cytotoxic, disrupt membrane integrity and form ion-channel pores.

## Introduction

Alzheimer’s disease (AD) accounts for at least two thirds of dementias, with currently 50 million sufferers worldwide^1^. AD is characterised by the accumulation of peptides cleaved from the amyloid precursor protein (APP), which form diffuse amyloid deposits and senile plaques in the brain^2^. Inherited forms of AD are caused by mutations in APP, or the γ-secretase responsible for APP cleavage, this makes a strong causal link between peptides cleaved from APP and Alzheimer’s disease^3–6^.

**Fig. 1:**
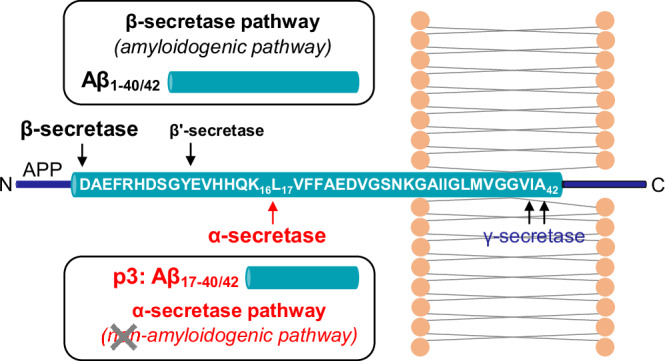
β- and α-secretase processing of amyloid precursor protein (APP). The amyloidogenic pathway, when Aβ is produced by the sequential cleavages of APP by β- and γ- secretases. The alternative pathway, mis-named as non-amyloidogenic, in which APP is cleaved by α-secretase between Lys16 and Leu17, to produce p3. γ-Secretase cleaves the C-terminus most commonly at position 40 or 42, within the lipid bilayer.

Aβ and p3 are endogenous peptides that are cleaved from the larger transmembrane amyloid precursor protein (APP) by the action of three enzymes, Fig. 1^5–7^. The β-secretase cleaves Aβ at its N-terminus. While the γ-secretase complex, cleaves APP, to form the C-terminus of Aβ. The cleavage point is variable but typically results in Aβ peptide 40 or 42 amino acids in length. An alternative cleavage pathway caused by the action of the α-secretase can result in a much shorter more hydrophobic peptide, named p3, which contains residues 17-40/42 of the Aβ sequence^8,9^. To distinguish it from the β-cleavage, this pathway has been mis-named ‘non-amyloidogenic’. Using cultured cortical neurons, it has been shown APP is cleaved by α-secretase twice as frequently as by the β-secretase^10^.

The p3 peptide is reported to be found in selected areas of the AD brain, in particular, diffuse amyloid deposits and at low levels in senile plaques^11,12^. The p3 peptides have also been identified in cerebrospinal fluid for AD patients^13^. While for those with Down syndrome, which is associated with a form of early-onset AD, the p3 peptide is dominant over full-length Aβ in pre-amyloid deposits of the cerebellum^14^.

Perhaps because of its designation as non-amyloidogenic and its poor solubility, there have been few biophysical studies of p3, relative to full-length Aβ. The contrast in publication rate is highlighted in Supplementary Fig. 1. For a careful review of these studies see; Kuhn and Raskatov^15^. Early studies on p3 report conflicting observations, with some studies indicating p3 does not form oligomers^16^ or fibrils^17^, while others have reported the opposite^18–20^. These discrepancies may be down to the difficulty in handling this poorly soluble peptide. More recent studies have confirmed p3_40_ can indeed form amyloid fibrils, while p3_42_ was found to be difficult to solubilize. Even in recent studies, the lag-phase in the kinetics of p3_40_ fibril formation was not observed, while the p3_42_ fibril growth kinetics were not studied^21,22^.

As both p3 and Aβ peptides are present in diffuse amyloid deposits and senile plaques, there is a possibility they impact each other’s fibril formation kinetics. Self-seeding is a well-established phenomenon, where preformed fibrils of the same Aβ peptide sequence nucleate fibril formation, this circumvents primary nucleation processes and reduces fibril formation lag-times^23,24^. While the ‘parent’ seeds often propagates the same structural morphology in the ‘daughter’ fibril^25^. Potentially cross-seeding, where different sequences of Aβ might nucleate fibril formation may also occur in vivo. Thus, a relatively small proportion of a highly amyloidogenic Aβ isoform, could have a major impact on fibril formation kinetics for other more abundant isoforms. There have been relatively few studies of this cross-seeding phenomena, despite its physiological relevance, and these have been largely restricted to studies of cross-seeding between Aβ_40_ and Aβ_42_^23,26–28^. It is becoming clear this process is very peptide specific^29–31^ and should not be confused with a less specific oligomer interaction before fibril formation^23^, or accumulation of plaques once fibrils are formed^32^. It is generally understood Aβ_40_ and Aβ_42_ do not cross-fibrillize but form fibrils independently^23,26–28^. The loss of just two amino acids from the C-terminus makes Aβ_40_ and Aβ_42_ fibril structures incompatible. Preliminary studies have suggested cross-seeding between p3_40_ and Aβ_40/42_^21,22^, while cross-seeding between p3_42_ and p3_40_ and the cross-seeding between p3_42_ and Aβ_40/42_ have yet to be explored. More widely, there are numerus examples of the interaction and cross-seeding between different amyloid forming proteins, which may impact fibril formation in-vivo^33–35^.

The mechanism by which Aβ and perhaps p3 triggers the cascade of events that leads to AD is not yet fully resolved, one popular hypothesis surrounds the action of Aβ on lipid membranes^36–38^. Aβ oligomers and curvilinear protofibrils carpet the lipid membrane inserting into the upper leaflet^36^, this disrupts the integrity of the membrane causing permeability and unregulated cellular Ca^2+^ influx^39,40^. A more specific Aβ interaction with the bilayer involves ring-shaped annular assemblies, these can insert into the membrane forming large unregulated ion-channel pores^41–45^. This allows flow of Ca^2+^ and other ions into the cell. This membrane permeability can then trigger a cascade of cellular events, including phosphorylation of tau, that culminates in a loss of neurons and dementia^3,4^.

Here we aim to characterise the assembly properties of the p3 peptides; Aβ_(17-40/42)_. We will investigate the cross-seeding kinetics of p3 with full-length Aβ peptides in vitro. Furthermore, we will probe the ability of the p3 peptides to disrupt lipid membranes and cause Ca^2+^ influx and cytotoxicity. We show p3 peptides form amyloid fibrils more rapidly than Aβ, and p3 can cross-seed and nucleate the fibril formation of full-length Aβ, but only for specific Aβ:p3 combinations with the same C-terminal length. The cytotoxic p3 peptides form annular oligomers, and p3_42_ oligomers form ion-channel pores across cellular plasma membranes. The misnaming of p3 as non-amyloidogenic has meant its possible role in Alzheimer’s disease has too often been underplayed. Indeed it is often suggested that elevating α-secretase activity might be protective by reducing levels of Aβ and so might be a therapeutic approach^9^, however here we show production of p3 rather than Aβ may in fact be undesirable.

## Results

### Fibril formation, p3 kinetics is dominated by secondary nucleation

**Fig. 2:**
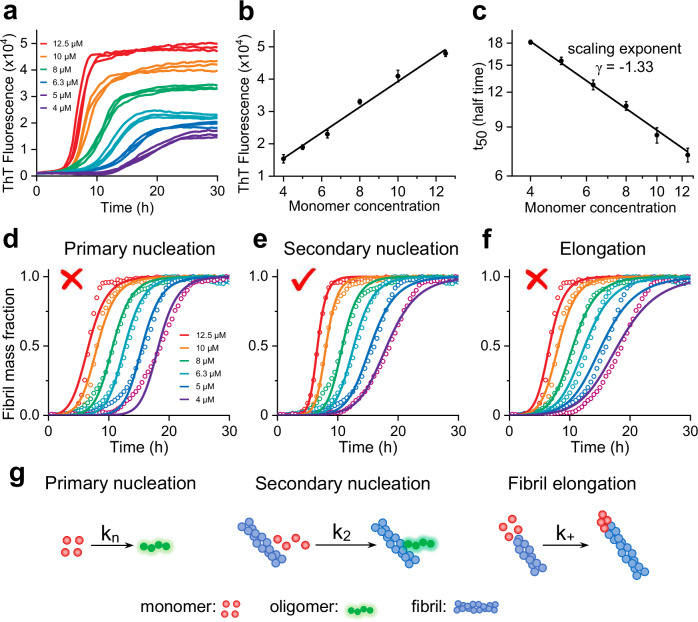
Concentration dependent aggregation of p3_42_. **a** Kinetics profiles of p3_42_ at initial monomer concentration from 4 μM (purple) to 12.5 μM (red) with individual technical replicates. **b** ThT fluorescence at the plateau-phase against the p3_42_ monomer concentrations. **c** Double logarithmic plot of half time (t_50_) versus the initial monomer concentrations, scaling exponent γ = -1.33. Error bars are standard error of the mean (SEM) from three replicates. **d**–**f** Global fits of the kinetic traces when only primary nucleation (**d**), secondary nucleation (**e**) and fibril elongation (**f**) rate constants are altered to globally fit concentration dependent traces. Kinetic traces fit well to secondary nucleation with a low mean residual error (MRE = 0.0009), while primary nucleation and fibril elongation fit less well (MRE = 0.0019; 0.0023 respectively). Global fitting indicates fibril formation is dominated by secondary nucleation. The ‘check’ and ‘cross’ marks on the charts refer to the kinetic traces fitting well or less well to the models. **g** Schemes of the microscopic steps for primary nucleation (k_n_), secondary nucleation (k_2_) and fibril elongation (k_+_).

The amyloid specific dye, thioflavin-T (ThT), has been used to monitor p3 fibril formation. In contrast to previous studies that were unable to obtain fibril growth kinetic curves, with a clear lag-phase for p3_40_ or p3_42_^21,22^, we have found by solubilizing p3_40/42_ peptide in aqueous solution, at pH 10, followed by size-exclusion chromatography (SEC) purification, to remove any remaining nucleating seeds, it was possible to obtain highly reproducible kinetic data for both p3_40_ and p3_42_ fibril formation. We show the kinetic traces exhibit sigmoidal growth-curves characteristic of a nucleation polymerization reaction and exhibit a clear lag- and elongation- phase, Fig. 2 and Supplementary Fig. 2. The loss of the many solubilizing hydrophilic amino acids, within residues 1–16, results in the p3 peptides forming fibrils much more readily than full-length Aβ, under the same conditions. Indeed, the reaction half-time, t_50_, are reduced by more than half, for the p3 peptides, Supplementary Fig. 2f. In contrast to previous reports^17,22^, we also show that p3 and Aβ fibrils produce comparable ThT fibril fluorescence intensities, for preparations with the same initial monomer concentration, Supplementary Fig. 2e.

There are numerous steps that describe the molecular processes of assembly into amyloid fibrils. Each step has its own micro-rate constants, associated with the macroscopic kinetic profiles^46,47^. It has previously been shown that full-length Aβ is dominated by secondary nucleation^46^, these processes are believed to involve the formation of nucleating oligomers on the surface of fibrils. We wondered if the p3_40/42_ peptides were also dominated by secondary nucleation.

We have monitored p3 fibril formation for both p3_42_ and p3_40_, over a range of initial monomer concentrations, between 4-12.5 µM, Fig. 2 and Supplementary Figs. 3, 4. Figure 2c shows the reaction half-time (t_50_) scales with the initial monomer concentration, under quiescent conditions. The scaling exponent (slope) are as follows: γ = -1.33 (p3_42_) and γ = -1.39 (p3_40_). These values are very similar to that reported for full-length Aβ_42_ (also γ = -1.33)^46^. The linear dependence of this double logarithmic plot, Fig. 2c, indicates saturation effects are not important over the concentration range we have used^46–48^. We have used the online fitting programme, ‘AmyloFit’^48^ to globally fit the macro-kinetic traces for both p3_42_ and p3_40_, over a range of concentrations for the whole reaction course, Fig. 2d-f and Supplementary Fig. 3. Here we show that p3 fibril formation kinetic processes are indeed also dominated by secondary nucleation(k_2_). This is where nucleating oligomeric seeds form on the surface of fibrils^47,49,50^. The kinetics is both monomer and fibril concentration dependent, resulting in sharp exponential growth in the kinetic traces, which is not so marked when only primary nucleation takes place, Fig. 2d.

### p3 assemblies, secondary structure and morphology

**Fig. 3:**
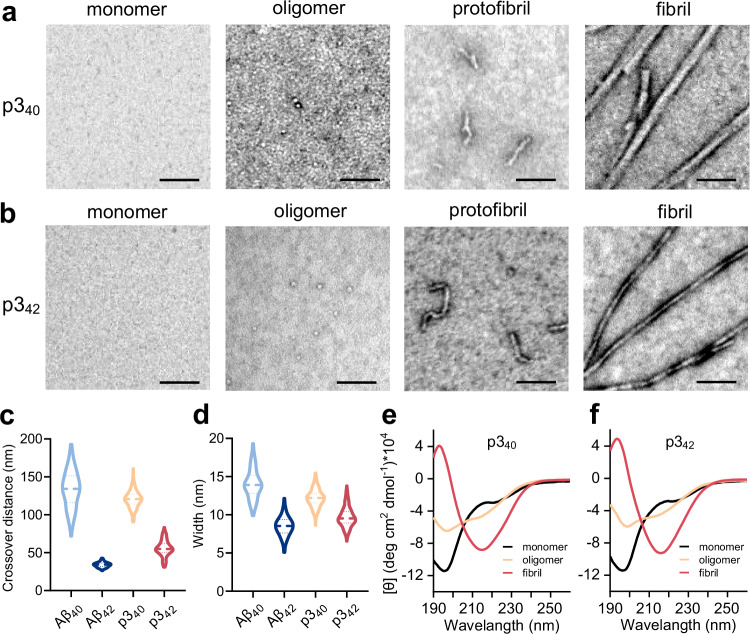
The self-assembly of p3_40_ and p3_42_. **a**, **b** TEM images of monomer, oligomer, protofibril and fibril of p3_40_ (**a**) and p3_42_ (**b**). The morphology of the p3 peptides were reproducible and consistent on at least three independent experiments. Scale bars: 50 nm. **c**, **d** Comparison of fibril twist periodicity (**c**) and fibril widths (**d**). Data suggest similar morphology for fibril with the same C-terminal truncation. **e**, **f** Far-UV CD spectra of p3_40_ (**e**) and p3_42_ (**f**) indicates transition in secondary structure from random coil (198 nm) to β-sheet (217 nm) for fibrils. Preparations (10 μM) were incubated in 10 mM sodium phosphate buffer, pH 7.4.

Transmission electron microscopy (TEM) has been used to image p3 peptides at different time points during fibril assembly. Sampling at the end of the lag-phase of fibril growth, the images reveal prefibrillar oligomeric and curvilinear protofibril structures, Fig. 3a, b. Additional, TEM images of oligomeric p3_40_ and p3_42_ are shown in Supplementary Fig. 5. After further incubation TEM images indicate fibrillar assemblies become widespread. The fibril morphology has a similar appearance to full-length Aβ. The twists in these fibrils, formed from SEC purified p3 peptide, under quiescent conditions, are compared to full-length Aβ fibrils, formed under the same conditions, Fig. 3c. Despite the loss of a third of the polypeptide chain, the fibril twist morphology of the p3 peptides are similar to the full-length Aβ counterparts, Fig. 3 and Supplementary Fig. 6. In particular, Aβ_40_ and p3_40_ have a similar pronounced twist periodicity, both have a long crossover distance within a range of between 101 and 166 nm for Aβ_40_ and 106–134 nm for p3_40_, Fig. 3c. While Aβ_42_ and p3_42_ counterparts have much tighter twists, with a crossover distance of just 30–38 nm and 41–71 nm, respectively. Furthermore, the range of fibril widths have a similar relationship. Aβ_40_ and the p3_40_ counterpart have similar fibril widths of 12–17 nm and 11–14 nm, while, Aβ_42_ and p3_42_ peptides have narrower widths of 7–10 nm and 8–12 nm, Fig. 3d. The mean fibril crossover distance and width are presented in Supplementary Table 1 for the four peptides. Histograms of the distribution of crossover distance and fibril widths are shown in Supplementary Fig. 7. The range of fibril twists indicates some polymorphism for each peptide^51,52^. However, the range of values clearly group together, with a relatively long helical crossover lengths for Aβ_40_ and p3_40_ (134 nm and 120 nm) and much shorter crossover lengths for Aβ_42_ and p3_42_ (34 nm and 56 nm), Supplementary Table 1. It remains to be established how similar the fold topology is between these p3 and Aβ fibrils.

Circular dichroism (CD) spectra are consistent with a cross-β structure for the fibrils of p3 peptides, Fig. 3e, f. The CD spectra of the p3 fibrils are dominated by a strong negative CD band, centred at 217 nm and a positive band at 198 nm. This indicates a high proportion of β-sheet present. The SEC purified monomer is dominated by a negative band at 198 nm, which is characteristic of irregular structure, while lag-phase oligomers show an increased proportion of β-sheet, but still retain many monomers with a random-coil ellipticity in the mixed solution.

### Cross-seeding between p3 and Aβ peptides

**Fig. 4:**
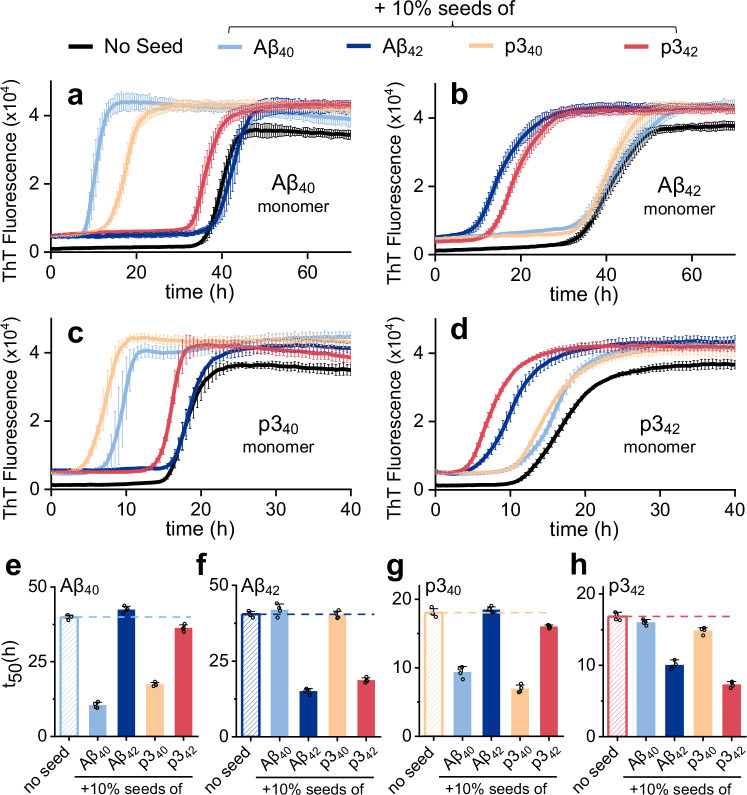
Aβ cross-seeding with p3 peptides. **a**–**d** Fibril formation of monomeric Aβ_40_ (**a**), Aβ_42_ (**b**), p3_40_ (**c**) and p3_42_ (**d**) in presence of a range Aβ isoform fibril seeds (10% w/w): No seed (black); Aβ_40_ (pale blue); Aβ_42_ (dark blue); p3_40_ (orange); and p3_42_ (red) seeds. **e**–**h** The mean t_50_ are shown for Aβ_40_ (**e**), Aβ_42_ (**f**), p3_40_ (**g**) and p3_42_ (**h**) fibril formation in presence of a range Aβ isoform fibril seeds. Error bars are SEM from four replicates. Kinetic curves represent mean from individual technical replicates.

**Fig. 5:**
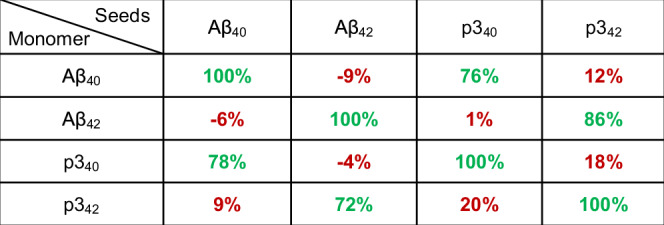
Tabulated t_50_ for cross-seeding. The *t*_*50*_ values for each combination of monomer with different seeds, presented as a percentage relative to self-seeding *t*_*50*_ values. Values are from the data shown in Fig. 4. and Supplementary Fig. 8 and Table 2. The values highlight profound seeding >70% for seeding between the p3_40_:Aβ_40_ and p3_42_:Aβ_42_ pairs, shown in green. In contrast, other combinations of peptides had a negligible impact on lag-times, shown in red. The relative impact on *t*_*50*_ values, expressed as a percentage, are calculated as follows: (Unseeded t_50_ - Cross seeded t_50_ **/** Unseeded t_50_ - Self seeded t_50_) x 100%.

The p3 and Aβ peptides are both released at the synapse and so may potentially influence the kinetics and morphology of each other’s fibril formation in vivo. The p3 peptides lack many of the solubilizing sidechains in the N-terminus of Aβ and consequently form fibrils much more rapidly, in half the time of full-length Aβ, under the same conditions, Supplementary Fig. 2. We wondered once p3 peptides form oligomers and fibrils, these assemblies might be capable of nucleating full-length Aβ fibril formation. In Fig. 4 and Supplementary Fig. 8, we show self- and cross- seeding experiments between p3 and Aβ. Very pronounced acceleration in fibril formation does indeed occur, with particular combinations of Aβ and p3 seeds. The nucleating interactions are very specific, with p3_40_ fibril seeds (1 µM, 10% p3_40_) nucleating Aβ_40_ monomer and reducing lag-times by almost half, while p3_40_ fibril seeds have almost no effect on the kinetics of p3_42_ or Aβ_42_, Fig. 4a. Similarly, only p3_42_ is capable of cross-seeding with Aβ_42_ monomers, Fig. 4b. This behaviour is precisely echoed with monomeric p3_40_ and p3_42_ while using fibril seeds of Aβ_40/42_, Fig. 4c, d. The self- and cross-seeding pattern is very apparent when tabulated in Fig. 5. This bi-directional nature of cross-seeding is highlighted by the mirror symmetry across the diagonal shown in Fig. 5. Self-seeding typically causes a halving of lag-times (using 1 µM, fibril seeds), but also a very similar reduction in t_lag_ and t_50_ times for cross-seeding, but only between the p3_40_:Aβ_40_ and p3_42_:Aβ_42_ pairs, Fig. 5, Supplementary Table 2. Other combinations of p3 with full-length Aβ have negligible impact on kinetics and do not cross-seed, Fig. 5. Also of note, there is minimal cross-seeding between p3_40_ and p3_42_, Fig. 5, this is analogous to the minimal cross-seeding reported between Aβ_40_ and Aβ_42_^22^.

We suggest the strong influence of p3_40/42_ on the complementary fibril forms Aβ_40_ or Aβ_42_, relates to the impact the two C-terminal residues have on fibril structure. In particular, it is well documented the addition of two amino-acids have a major influence on the fundamental fold of Aβ_40_ and Aβ_42_. The two isoforms typically form an ‘U-shaped/extended’ and a ‘S-shaped’ folds respectively, these are apparent in many of the fibril structures reported under a variety of conditions^53–58^. The extension by two amino acids at the C-terminus, allows the stabilisation of the S-shaped fold, in which a salt-bridge is formed between the Ala42, C-terminal carboxylate, and the amino group of Lys28. This is not possible in Aβ_40_ and so a salt-bridge is formed between Lys28 and Asp23^55,56^. The cross-seeding between p3 and Aβ suggests the C-terminus is fundamental to influencing the fold within fibrils and thus the compatibility between nucleating seeds. While the loss of residues 1–16 have less of an impact on the fold. We suggest that the structure of p3_40_ and p3_42_ will show similar U- and S-shaped topologies respectively. Although atomic details are yet to be resolved for the p3 peptides, the similarities in structure to the full-length Aβ_40_ and Aβ_42_ counterparts is suggested by the similarity in fibril twist periodicity and widths, shown in Fig. 3c, d and Supplementary Table 1.

It is notable the reduction in lag-times observed for cross-seeding is comparable in magnitude to the Aβ and p3 self-seeding kinetics, perhaps suggesting that the molecular processes are similar. It remains to be established if the cross-seeding phenomena is driven by fibril surface-catalysed secondary-nucleation or templating from the ends of seeding fibrils. In the case of self-seeding both lateral surface-catalysed secondary-nucleation and templating elongation occurs. Koloteva-Levine et al reports an excellent analysis of these two molecular behaviours^59^.

We have previously studied a different N-terminal truncated form of Aβ; Aβ_11-40/42_, formed from an alternative cleavage site of the β-secretase^29^. Like the pattern of cross-seeding for the p3 peptides in this study, we also showed that cross-seeding can occur only between Aβ_40_ and Aβ_11-40_, while Aβ_11-42_ is only compatible with Aβ_42_ and will cross-seed and cross-fibrilize^29^. A pattern of behaviour is emerging for this very specific cross-seeding behaviour. Deletions of residues 1–10 or 1–16 have little impact on the compatibility between Aβ peptides. While the C-terminal truncation of just two residues impacts the ability for Aβ to cross-seed. We have recently shown compatibility between fibril cross-seeding of Aβ_42_ with the Arctic mutant Aβ_40_(E22G), this is also associated with the U- and S-shaped fold, linked with the salt-bridge at Asp23-Lys28^30^.

### p3 membrane interaction and cellular homeostasis

**Fig. 6:**
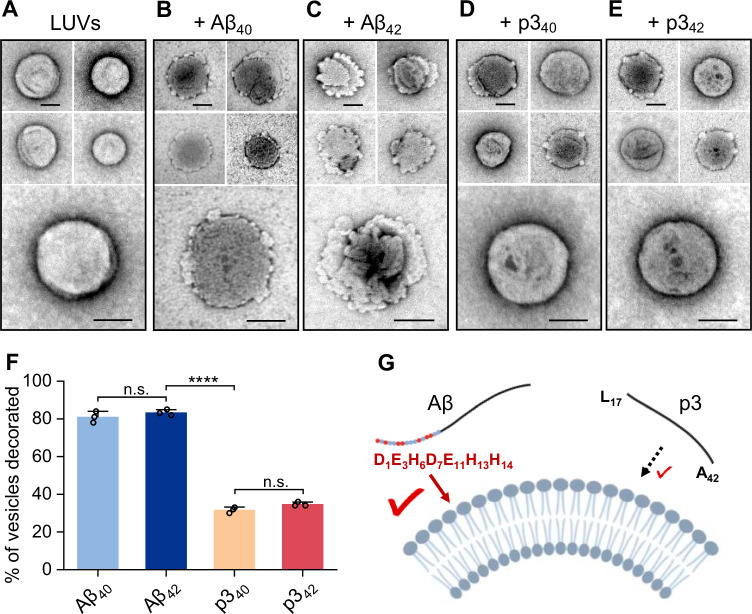
Impact of Aβ_40_, Aβ_42_, p3_40_ and p3_42_ oligomers on lipid vesicles. **A**–**E** Large unilamellar vesicles (LUVs) in the absence of Aβ (**A**); incubated with: (**B**) Aβ_40_ oligomers, (**C**) Aβ_42_ oligomers, (**D**) p3_40_ oligomers and (**E**) p3_42_ oligomers. Aβ and p3 peptide oligomers are taken from the end of the lag-phase after incubation of 10 µM monomeric preparations. Scale bar: 100 nm. More examples in Supplementary Fig. 9,10. **F** Analysis of lipid vesicles impact of Aβ oligomers. Error bars are SEM from three independent replicates, 100 vesicles imaged per replicate. One-way ANOVA test, the symbols n.s. and ****, indicate *P-*values of Aβ_40_-Aβ_42_, *P* = 0.49; p3_40_-p3_42_, *P* = 0.30; and ****P ≤ 0.0001, p3_42_-Aβ_42_, respectively. **G** Cartoon highlighting loss of charged residues causes a reduced interaction with the phospholipid bilayer.

It is clear p3 peptides are capable of forming prefibrillar oligomeric and curvilinear protofibril structures, Fig. 3a,b. We therefore wondered if they might interact with lipid bilayers and cause membrane permeability, similar to that reported for full-length Aβ^36,37^. We have used large unilamellar vesicles (LUVs) and imaged the interaction of p3 peptides with lipid bilayers, and then compared the affects with their full-length Aβ counterparts. In Fig. 6, we show a series of typical LUVs, imaged by TEM, incubated in the presence of Aβ or p3 peptides. Lipid vesicles in buffer, negatively stained with uranyl-acetate, have a largely circular (spherical) appearance, with a relatively smooth membrane surface, Fig. 6a and Supplementary Fig. 9, 10. Oligomers and curvilinear protofibrils taken from the end of the lag-phase of Aβ_40_ and Aβ_42_ assembly (starting from 10 µM of peptide monomers), produced marked disruptions in the membrane surface, causing discontinuity and distortions in the membrane, Fig. 6b,c and Supplementary Fig. 9b, c. Inspection of >300 individual vesicles indicate >80% of the lipid membrane surface are disrupted in this way, Fig. 6f and Supplementary Table 3. We have previously shown Aβ_40_ and Aβ_42_ fibrils, or monomers, have little impact in lipid membrane integrity^36^. Here we show the p3 peptides, p3_40_ and p3_42_ oligomers, also disrupt lipid vesicles, but this is less widespread, Fig. 6d, e. Inspection of >300 vesicles suggests 32% and 35% of vesicles are disrupted, Fig. 6d-f, Supplementary Fig. 10 and Supplementary Table 3. This data implies a reduction in the impact p3 oligomers have on the lipid bilayers.

**Fig. 7:**
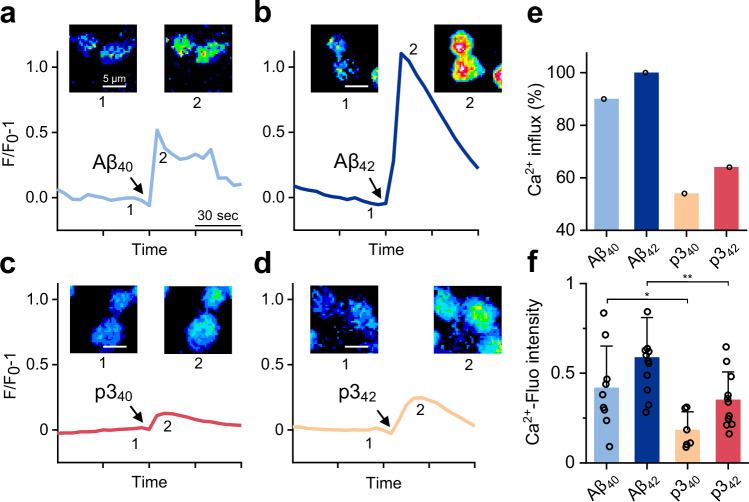
Calcium influx of HEK293T cells in response to different Aβ and p3 oligomer preparations. **a**–**d** Time-lapse recording of fluorescence relative to fluorescence before addition of (**a**) Aβ_40_ oligomer, (**b**) Aβ_42_ oligomer, (**c**) p3_40_ oligomer and (**d**) p3_42_ oligomer. Also shown are single-cell florescence images before (labeled 1) and after (labeled 2) the addition of Aβ or p3 oligomer. **e** Percentage of preparations showing Ca^2+^ influx. **f** Mean Ca^2+^ fluorescence intensity, (F/F_0_)-1, where F is the observed fluorescence and F_0_ is fluorescence just before addition of Aβ peptides. Error bars are SEM from six replicates. Two tailed pair-wise *t*-test, the symbols * and **, indicate *P-*values of 0.038 and 0.008, respectively.

Next, we wanted to investigate to what extent the presence of the p3 peptides can disrupt the cellular membrane and allow Ca^2+^ and other ions to flow into the cell lumen. For this study we chose HEK293 cells, which have neuronal character, and match the cell-line used for the well-established patch-clamp studies described later. Using a Ca^2+^ sensitive fluorescent dye, Fluro-3, we have monitored the impact of Aβ and p3 peptides on cellular membrane permeability. In agreement with the TEM images, Aβ_40_ and Aβ_42_ oligomers cause influx of Ca^2+^ into the cell, within 30 s after addition of Aβ, for almost 100% of the preparations studied, Fig. 7a, b. Previous studies have shown Aβ_42_ induced Ca^2+^ influx is from an extra-cellular source, not from intra-cellular stores^40^. The p3 oligomers cause cellular Ca^2+^ influx less frequently, with 54% and 64% of cell preparations having detectable Ca^2+^ influx, for p3_40_ and for p3_42_, respectively, Fig. 7c-e. Furthermore, for preparations that do exhibit Ca^2+^ influx, the fluorescence intensity is also reduced, Fig.7f. The HEK293 cells have less Ca^2+^ influx when exposed to p3 oligomers relative to Aβ. This supports the assertion that the N-terminal residues, although not essential for lipid disruption, do enhance the level of interaction with the lipid bilayer.

**Fig. 8:**
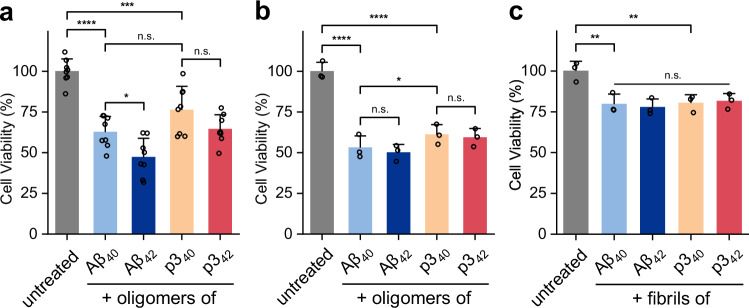
Cell viability after treatment with Aβ or p3. **a** Cytotoxicity of oligomers on rat primary neurons. **b**-**c** Cytotoxicity of oligomers (**b**) and fibrils (**c**) on HEK293 cells. The cells were incubated for 24 h with oligomers or fibrils. Aβ_40_ (pale blue), Aβ_42_ (dark blue), p3_40_ (orange) and p3_42_ (red). Replicate measurements were *n* = 8 and *n* = 3 for primary neurons and HEK293 cells respectively. Error bars indicate standard error of the mean. One-way ANOVA test (normal distribution confirmed with Shapiro-Wilk test), the symbols n.s., *, **,*** and ****, indicate *P-*values of (**a**) Aβ_40_-p3_40_ *P* = 0.11; p3_40_-p3_42_ *P* = 0.21; *P = 0.0484; ***P = 0.0008 and ****P ≤ 0.0001 (b) Aβ_40_-Aβ_42_ *P* = 0.97; p3_40_-p3_42_ *P* = 0.99; *P = 0.50 and ****P ≤ 0.0001; (**c**) control-Aβ_40_ **P = 0.007 and control-p3_40_ **P = 0.008. Aβ and p3 preparations were from 10 µM monomer equivalent, the controls were cells treated with an equal amount of buffer. Cell viability, monitored by MTT essay, indicates that Aβ and p3 oligomers were cytotoxic to rat primary neurons and HEK293 cells.

Cell viability measurements support the differential effects of Aβ and p3 on membrane permeability, Fig. 8. We studied both rat primary neurons, Fig. 8a, and HEK293 cells, Fig. 8b, c. Assessment of cell viability, measured using an MTT assay, suggests that Aβ_42_ oligomers are the most cytotoxic form, followed by Aβ_40_. The p3_42/40_ oligomers were also cytotoxic but less so than Aβ_42_ oligomers, Fig. 8a, b. Cell treatment with fibril preparations of Aβ and p3 result in less cytotoxicity, with only a slight reduction in cell viability relative to treatment with the buffer control, Fig. 8c.

Aβ contains most of its charged residues in the N-terminus, residues 1–16, with four carboxylate sidechains as well as three charged histidine side-chains. The loss of these in the p3 peptide will mean less interaction with charged phospholipid head-groups. An initial electrostatic interaction with the residues in the N-terminus of Aβ, followed by insertion into the membrane, is suggested as a mechanism for Aβ interactions with lipid membranes^60,61^.

### p3 annular ring assemblies

It is postulated that Aβ cytotoxic action is caused by its ability to form annular structures, that insert into the membrane and form large unregulated ion-channel pores^41–44^. We wondered if p3 oligomers could form these ion-channels, and what impact the loss of the N-terminal residues (carboxylates and histidine’s) would have on the ion-channel properties.

**Fig. 9:**
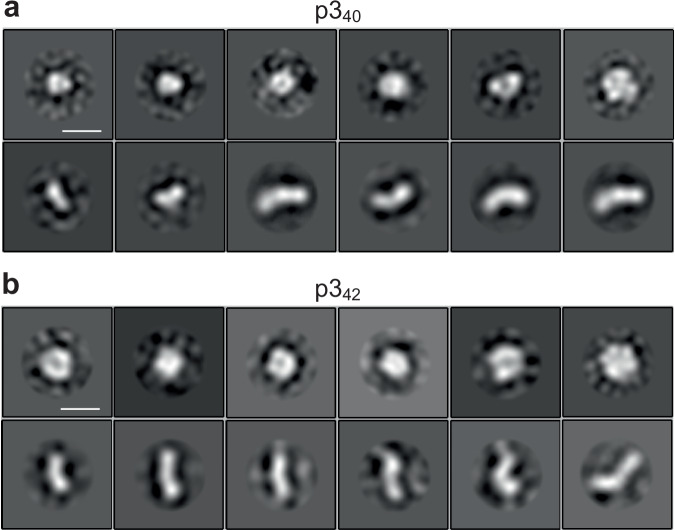
2D class averages of oligomers and curvilinear protofibrils for (a) p3_40_ and (b) p3_42_. *Ca*. 150 single particles for each of the 2D class averages. Top rows show annular ring structures for both p3_40_ and p3_42_. While bottom rows show curvilinear protofibrils with consistent widths. Oligomers are negatively stained with uranyl acetate. Scale bar: 10 nm.

Analysis of TEM images of p3_40_ and p3_42_ peptides, taken at the end of the lag-phase, reveal a snapshot of prefibrillar assemblies. Curvilinear protofibrils, but also many oligomeric structures that resemble annular ring-like assemblies, are widespread in the micrographs. Typical micrographs are shown in Supplementary Fig. 5. The p3 annular structures, shown in Fig. 9, have a very similar appearance to those reported for Aβ_42,_ which we have recently studied by cryo-EM and cryo-ET^45^. We have used single particle analysis to generate 2D class averages for both p3_40_ and p3_42_ oligomers, Fig. 9. Particularly noteworthy are the class averages, which have a ring-shaped appearance. Dimensions of the external ring are *ca*. 7–10 nm with an internal channel ~1–2 nm in diameter. These annular assemblies of p3 have not previously been reported and add to the number of amyloid forming proteins that also form annular structures^62^. The curvilinear protofibrils, also apparent, have a consistent width. These have been measured to be 2.8 nm, recently characterised for Aβ_42_ by cryo-EM and cryo-ET^45^. Despite the loss of residues 1–16, the p3 peptides are capable of forming curvilinear protofibrils and annular structures that are very similar to those reported for full-length Aβ^45^. Residues 1 to 14 have been shown to be very flexible in fibrils, with a lack of stable hydrogen-bonds in this region^63^. These N-terminal residues may therefore not contribute to the stability of fibrils or pre-fibrillar assemblies.

### Ion channel pore formation

**Fig. 10:**
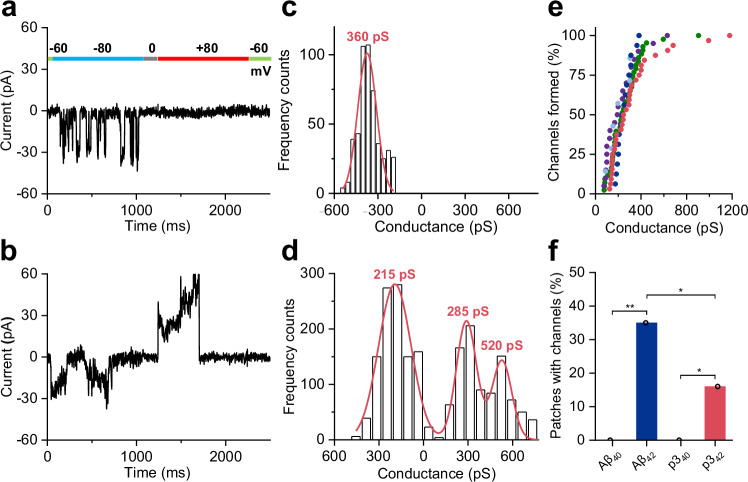
Ion channels of p3_42_ oligomers. **a**, **b** Patch-clamp current recordings, 2.5 s, across HEK293 cell membrane patches, with extra-cellular p3_42_ oligomers (5 μM). **c**, **d** Frequency distribution of conductance for the same traces, gaussian curves indicate conductance values. On the left conductance values when a negative potential was applied (-80 mV), whereas on right conductance value are when the applied potential was positive ( + 80 mV). **e** Range of conductance observed for p3_42_ for five individual membrane patches. **f** Percentage of membrane patches that record ion channels for Aβ and p3 oligomers. Fisher test, the statistical test used was two-sided, the symbols * and ** indicate *P*-values of Aβ_40_-Aβ_42_ **P = 3 × 10^-7^; p3_40_-p3_42_ *P = 0.05; Aβ_42_-p3_40_ *P = 0.06.

To study the ion-channel forming properties of the p3 peptide, we used excised membrane patches from HEK293 cells and performed patch-clamp electro-physiology measurements. Here we have used an ‘inside-out’ configuration to allow p3 peptides to diffuse to the extracellular surface of HEK cell membranes. We then measured channel conductance across the lipid-bilayer using a stepped voltage of -80, 0 and +80 mV, typical examples of which are shown in Fig. 10 and Supplementary Fig. 11. Large conductances, of *ca*. 200 pS for p3_42_ peptide are observed, these have a flickering appearance, rapidly opening and closing, Fig. 10a, b. They are present in both, polarising and depolarising potentials. The ability of the channels to almost instantly fully close and open, suggests these conductance traces are typically for single channels. The size of the conductances can be related to channel pore-size formed by p3 annular oligomers. A 2,500 ms measurement (in pA) are converted to conductance (pS) and plotted as a histogram to reveal the range of conductance values, Fig. 10c, d. These have been fitted to a gaussian curve so as to obtain a typical conductance value for each 2500 ms recording.

The range of conductances is quite variable, Fig. 10e, typically between 150–355 pS (20–80% of the total range). This range of conductance values are similar to Aβ_42_, previously reported,^44,45^ with conductance values ranging between 200–575 pS, Supplementary Fig. 12. The loss of residues 1–16, including a number of charged residues, does not markedly affect the conductance properties of the channel pores, although median conductance values are a little smaller, by 140 pS. Assuming a channel length of 54 Å (the lipid bilayer thickness) these conductance values suggest a channel diameter of 1.1 to 1.5 nm^44,45^. This is in-line with the internal diameter of the annular structures shown in Fig. 9 for p3_42_.

Aβ_42_ oligomers produce channels in excise cellular membranes for 35% of patch-clamp recordings^44^. The numbers of channel conductance observed for p3_42_ is less, five pulled patches produced channels from 32 recordings (16%), Supplementary Table 4. The reduction in the number of channels formed, 16% compared to 35% for full-length is significant (*P* < 0.1, Fisher test). The reduced interaction of p3_42_ with the membrane is also suggested from the vesicle imaging, Fig. 6, and Ca^2+^ influx measurements, Fig. 7. The loss of sixteen N-terminal residues reduces the interaction with the membrane.

Importantly, we show only p3_42_ peptide inserts into cellular membranes and forms channels but not p3_40_ (Fig. 10, Supplementary Fig.11, Supplementary Table 4). This mimics the behaviour of full-length Aβ_40_ and Aβ_42_, where only Aβ_42_ forms a large conductance across the membrane, Fig. 10f. This significant distinction in channel forming properties of Aβ_40_ and Aβ_42_ is important, as it parallels the pathology of the two peptides; Aβ_42_ is thought to be the pathogenic isoform while Aβ_40_ may be harmless^64–66^. The channel forming ability of p3_42_, but not p3_40_, supports the assertion that the Aβ_42_ peptides pathogenicity is link to its ion-channel forming ability^44^.

The membrane conductance recorded appear with a median time of *ca*. 5 min after exposure to p3_42_ oligomers. Presumably it takes this time for the annular oligomers to insert and rearrange within the membrane. Full-length Aβ_42_ also takes a number of minutes for the conductance to appear, with a median time to produce channels of 8 min^44^. The time for the ion channels to appear is distinct from the more general membrane permeability, measured by Ca^2+^ influx, Fig. 7, which occurs almost instantly after exposure to Aβ_40/42_ or p3_40/42_ peptides.

To conclude, as previously highlighted by Raskatov, the p3 peptides have been misnamed and are in fact highly amyloidogenic^21,22^. We suggest that the cleavage pathway of Aβ and p3 should be referred to as the β- and α-secretase pathways, rather than amyloidogenic and non-amyloidogenic, to avoid confusion, Fig. 1. Our cross-seeding studies suggest rapid fibril formation of both p3_40_ and p3_42_ can trigger Aβ oligomer formation in vitro. The cross-seeding behaviour is highly specific and occurs only when the C-terminal lengths are matched. With the presence of the p3 peptide identified in diffuse amyloid deposits from AD brains, it is conceivable this cross-seeding behaviour may occur in vivo, further studies, using brain-derived p3 fibrils, will be needed in the future. The p3_40/42_ peptides are capable of forming cytotoxic oligomers, curvilinear-protofibrils and annular structures. Like Aβ, these pre-fibrillar assemblies can cause membrane disruption, permeability and ion-channel pores in their own right. A role in AD pathology for this under-studied, hydrophobic, N-terminally truncated form of Aβ should certainly be considered.

## Methods

### Ethical Statement

The cultures were produced using tissue from Sprague Dawley rat pups. Dams (strain code: CD(SD)) were obtained from the breeder (Charles River, UK), and maintained and humanely culled in accord with the UK Animals (Scientific Procedures) Act 1986 Amendment Regulations 2012 (SI 2012/3039) and the EU Directive 2010/63/E.

### Aβ and p3 peptides

All synthetic Aβ peptides were purchased from EZBiolab Inc in a lyophilized form. Aβ peptides were synthesized by solid-phase F-moc (N- (9-fluorenyl)methoxycarbonyl) chemistry, and were purified with reverse-phase high performance liquid chromatography, Supplementary Fig. 13. Sequences was verified by electrospray ionization mass spectrometry (ESI-MS), Supplementary Fig. 13. ESI-MS measurements were performed on Agilent 6125 LC/MSD mass spectrometer (Agilent Technologies, Santa Clara, CA, USA). Peptides were desalted using a desalting column and samples were directly injected. Data were recorded in positive mode in an m/z range of 200 – 2000. Data were processed using MSD Chemstation software (Agilent Technologies). The following amino acid sequences were generated:

Full-length Aβ_40_: DAEFR HDSGY EVHHQ KLVFF AEDVG SNKGA IIGLM VGGVV

Full-length Aβ_42_: DAEFR HDSGY EVHHQ KLVFF AEDVG SNKGA IIGLM VGGVV IA

p3_40_ Peptide Aβ_17-40_: LVFF AEDVG SNKGA IIGLM VGGVV

p3_4**2**_ Peptide Aβ_17-4**2**_: LVFF AEDVG SNKGA IIGLM VGGVV IA

Unless otherwise stated, all other chemicals were purchased from Sigma-Aldrich and ThermoFisher scientific.

### Aβ and p3 solubilization and isolation

The lyophilized full-length Aβ and p3 peptides were solubilized in ultra-high quality water to 0.7 mg ml^-1^ and adjusted to pH 10 with NaOH and left at 4 °C for 30 min. Then, the peptide solutions were centrifuged for 15 min at 20,000 g at 4 °C, to remove any high molecular weight aggregates. Crucially, size-exclusion chromatography (SEC) was used to remove any remaining nucleating and oligomeric aggregates for generating a seed-free preparation described as monomeric. Prefibrillar samples of Aβ and p3 peptides described as oligomeric were obtained as a heterogeneous mixture from the end of the lag-phase, as monitored by ThT in adjacent wells. These oligomeric preparations contain a range of curvilinear protofibrils and annular assemblies as well as an appreciable number of peptide monomers. Fibril samples were taken at the plateau-phase of fibril formation.

### Size exclusion chromatography (SEC)

Monomeric Aβ was isolated using AKTA FPLC with a Superdex 75 10/300 GL column (volume = 24 ml; GE Healthcare) at a flow rate of 0.5 mL·min^−1^ at 4 °C. The column was pre-equilibrated with 30 mM sodium phosphate buffer (pH 7.4). The wild-type Aβ_40_ and Aβ_42_ peptide concentrations were determined using the single tyrosine absorption at 280 nm, ε_280_ = 1280·cm^−1^ mol^−1^. p3_40_ and p3_42_ sequences lack a tyrosine residue (Tyr10), so peptide concentrations were determined by the amide absorbance at 205 nm (ε_205_ = 83370 M^−1^ cm^−1^ for p3_40_ and ε_205_ = 88930 M^−1^ cm^−1^ for p3_42_) using an online protein calculator^67^. Negative-stain electron microscopy and thioflavin-T fluorescence assay confirmed that SEC-purified peptides were seed-free. Monomeric peptides were used directly after SEC, Supplementary Fig. 14.

### Fibril growth assay

The kinetics of amyloid fibril formation were monitored with thioflavin T (ThT), a fibril-specific fluorescent dye^68^. Monomeric Aβ or p3 peptides (10 μM) with ThT (20 μM) were placed in a 96-well plate (200 μL per well) in 30 mM sodium phosphate buffer (pH 7.4) at 30 °C. The 96 well-plates were clear flat-bottom polystyrene, non-pyrogenic, non-tissue-culture treated plates (Corning 3881, USA). The well-plate remained quiescent throughout. ThT fluorescence was recorded by a FLUOstar Omega microplate reader (BMG Labtech, Aylesbury, UK) using the FLUOstar Omega software package, with excitation and emission filters at 440 nm and 490 nm respectively. Fluorescence readings were taken every 30 min. In the seeded aggregation assay, fibrils seeds (10%, 1 μM, Aβ or p3) were obtained by incubating 10 μM Aβ peptides in 30 mM sodium phosphate buffer at 30 °C for 3 days. Samples also contained DMSO 0.5% (0.5 mL/100 mL) used to solubilize ThT. The formation of Aβ and p3 fibrils was confirmed by ThT fluorescent assay and TEM imaging.

### Fitting fibril growth curves

The empirical kinetic values for t_lag_ (typically the time required for the ThT fluorescence to reach 10% of the maximum value) and, t_50_ (time required for half maximal fluorescence reached) were extracted from the data by fitting the fibril growth curve to the following equation^69^.

1 $$Y=\left({y}_{i}+\,{m}_{i}x\right)+\frac{{\upsilon }_{f}+{m}_{f}x}{{1+e}^{-(\frac{x-{xo}}{\tau })}}$$

Where Y represents the ThT fluorescence intensity, and x represents the time. x_o_ represents the time at which half maximal ThT fluorescence intensity is reached, referred to as t_50_. The lag-time (t_lag_) is taken from t_lag_ = X_0_ - 2τ. The initial and final fluorescence signals is represented by y_i_ and γ_f_, respectively^69^.

### Analysis of p3 peptide aggregation kinetics with AmyloFit

Global kinetic analysis of amyloid formation was preformed using the AmyloFit platform^48^. The amyloid fibril formation traces are described by the following integrated rate law, based on Michaelis-Menten-like kinetics:

2 $$\frac{M}{M({\infty })}=1-\left(1-\frac{M(0)}{M({\infty })}\right){{{\rm{e}}}}^{-{k}_{{\infty }}t}\times {\left(\frac{{B}_{-}+{C}_{+}{e}^{\kappa t}}{{B}_{+}+{C}_{+}{e}^{\kappa t}}\times \frac{{B}_{+}+{C}_{+}}{{B}_{-}+{C}_{+}}\right)}^{\frac{{K}_{{\infty }}^{2}}{{\bar{k}}_{{\infty }}\kappa }}$$

where the additional coefficients are functions of κ and λ:

3 $${C}_{\pm }=\frac{{k}_{+}{\left[P\right]}_{0}}{\kappa }\pm \frac{{k}_{+}M(0)}{2m(0){k}_{+}}\pm \frac{{\lambda }^{2}}{2{\kappa }^{2}}$$

4 $${k}_{{\infty }}=2{k}_{+}P({\infty })$$

5 $${\bar{k}}_{{\infty }}=\sqrt{{k}_{{\infty }}^{2}-2{C}_{+}{C}_{-}{\kappa }^{2}}$$

6 $${B}_{\pm }=({k}_{{\infty }}\pm {\bar{k}}_{{\infty }})/2\kappa$$

which are two combinations of the microscopic rate constants of:

7 $$\lambda=\sqrt{2{k}_{+}{k}_{n}{m(0)}^{{nc}}}$$

8 $$\kappa=\sqrt{2m(0){k}_{+}\frac{{m(0)}^{n2}{k}_{2}}{1+{m(0)}^{n2}/{K}_{M}}}$$

where *m*(0) represents the initial monomer concentration, *M*(0) represents initial fibril mass concentration, *M*(∞) represents mass concentration of fibrils at equilibrium, *P*(0) represents the initial aggregate concentration and *P*(∞) represents the aggregates concentration at equilibrium. The microscopic rate constants for primary nucleation (*k*_n_), secondary nucleation (*k*_2_), and elongation rate (*k*_+_). The exponents *n*_*c*_ and *n*_*2*_ are the reaction orders for primary and secondary nucleation respectively. *K*_*M*_ is the saturation constant for secondary nucleation. n_c_ and n_2_ were set as global constant of 2, so as not over-fit the data.

Wild-type Aβ_40_ and Aβ_42_ fibril assembly was fitted to a secondary nucleation model. The experimental macro kinetic traces were globally fitting to the integrated rate law over the range of p3 peptide concentrations. The microscopic rate constants *k*_+_*k*_n_; *k*_+_*k*_2_; and *k*_+_ values were fitted to the fibril growth curves at p3 peptide concentrations of 12.5 μM, the other kinetic traces at decreasing concentrations, were then fitted in three scenarios in which only one of the rate constants were permitted to vary, while the other two remain constant. This approach has been used to investigate how increasing concentrations of an inhibitor of fibril formation effect individual microrate constants^70^.

### Circular dichroism spectroscopy

Circular dichroism (CD) experiments were performed on an Applied Photophysics Chirascan instrument. CD spectra were obtained using a cuvette with a 1 mm pathlength, at 25 °C, under a nitrogen atmosphere. Spectra were recorded in the range of 190 nm – 260 nm with sampling points every 0.5 nm. Each spectrum represents the average of three scans. Data were processed using Applied Photophysics Chirascan Viewer and Origin software. The molar ellipticity [θ] (deg.cm^2^.dmol^-1^) were calculated using the equation [θ] = mdeg/ (l c), where ‘l’ is the pathlength and ‘c’ is the molar concentration.

### Transmission electron microscopy

Aβ and p3 peptide samples were generated with the same protocol used in the fibril growth assay, but without ThT addition. Oligomer samples were taken at the end of the lag-phase during fibril formation, while fibril samples were taken at the equilibrium phase. Aliquots (5 μL) of peptide samples (10 µM peptide) were added to carbon-coated 300-mesh copper grids, after being glow discharged (Agar Scientific Ltd, Essex, UK). The grids were then blotted after 90 s and rinsed with ddH_2_0 at room temperature. Uranyl acetate (5 μL of 2 mg/100 ml) was added, then blotted and rinsed after 10 s. Glow discharge was carried out using the Pelco EasiGlow unit. Images were recorded by a JEOL JEM-1230 electron microscope (JEOL Ltd., Japan) at 40,000 magnifications, operated at 120 kV, paired with a 2k Morada CCD camera and corresponding Olympus iTEM software package (Olympus Europa, UK). Crossover distances and fibril widths were measured by the segmented-line mode of the image-J software.

### Single particle analysis

Single particles were manually picked from 100 negatively stained TEM micrographs. 2D class-averages, for each preparation were generated, using RELION-4.1 software. CTFFIND-4.1 program was used to estimate the contrast transfer functions (CTFs) of the images. Approximately 2500 particles were manually picked from each of the datasets and the particles were extracted with a 31 nm box size. This was followed by a 2D classification into 20 classes, employing a mask with diameter of 200 Å. Representative 2D classes of annular oligomers and curvilinear protofibrils are shown in Fig. 9.

### Vesicle preparation

Large unilamellar vesicles (LUVs) were prepared using an extrusion method described previously^36^. The lipids used were egg phosphatidylcholine (PC) dissolved in chloroform, cholesterol dissolved in chloroform and the mono-sialotetrahexosl-ganglioside (GM1) dissolved in ethanol, at a ratio of 68:30:2 (by weight). Lipid solutions were placed in a fume hood overnight, to allow the organic solvent to evaporate. The lipid film was then re-solubilized in 30 mM sodium phosphate aqueous buffer (pH 7.4), to a lipid concentration of 1 mg ml^-1^. LUVs were generated using a benchtop mini extruder (Avanti Polar Lipids, Alabama, USA) at 60 ^o^C. The lipid solution was passed across a 100 nm polycarbonate membrane for 21 times. Lipids were stored at 4 °C for a maximum of 2 days before use.

### Cellular Ca(II) influx

Fluo3-AM loaded HEK293T cells: HEK293T cells were incubated at 37 °C, in 5% CO_2_ containing Dulbecco’s modified Eagle’s medium (DMEM, purchased from Thermofisher) supplemented with 10% foetal bovine serum and 0.2 mg ml^-1^ penicillin-streptomycin. Cells were plated into a 12-well plate (1 mL each well) incubated overnight, and cells were grown to 70–80% confluence. Next, cell media was replaced with fresh DMEM containing 5 μM Fluo3-AM (Abcam). To allow the cellular uptake of the Ca^2+^ sensitive fluorescent dye, plates were left in an incubator for a further 30 min. The cells were then washed twice with 400 μL of DMEM Eagles cell-media in each well, to remove the extracellular Fluo3-AM. Cells were incubated for a further 20 min at 37 °C, to allow de-esterification of intracellular AM esters, which activates intra-cellular Ca^2+^ dependent fluorescence. Finally, the DMEM was replaced with an aqueous buffer containing CaCl_2_ (1.8 mM); NaCl (120 mM); CsCl (10 mM); HEPES (9 mM); KCl (2.2 mM); and MgCl_2_ (1.9 mM), buffered to pH 7.4. The Fluo-3 loaded cells will then be ready for time-lapse fluorescence microscopy imaging.

Ca^2+^ Fluorescence Imaging of Fluo3: Fluorescence microscopy was performed using an inverted Leica DM IL microscope at 10x magnification. The bandpass filter allowed excitation at 470 nm, and emission was recorded at 520 nm. A charge-coupled device (CCD) camera was used to acquire time-lapse fluorescence images and bright-field visible light images, with a temporal resolution of one image every 5 s, recordings were for typically 9 min.

Aβ (Aβ_40_ oligomers; Aβ_42_ oligomers) and truncated Aβ peptide-p3 (Aβ_17-40_ oligomers; Aβ_17-42_ oligomers) were studied. Peptides samples with prefibrillar oligomers and curvilinear protofibrils were taken during fibril assembly at the end of the lag-phase. The 30 µM Aβ stock solutions was added to HEK293 cells within 400 µL buffer to produce 5 μM final Aβ concentration. Images were acquired using the ProgRes CapturePro 2.8.8 software and fluorescence intensity was measured by Image-J software. The time series analyser, V3 plug-in, was used to measure fluorescence intensities by analysing the overall field. We plotted (F/F_0_)-1 as the change in Fluo-3 fluorescence signals with time, where (F) represents the observed fluorescence and (F_0_) represents the background fluorescence at a time point just before the addition of Aβ or p3 peptides. Typically, *N* = 10–14 preparations with three independent repeats were obtained. Addition of Ionomycin, which is known to cause pronounced Ca(II) influx, and was used as a positive control. Ionomycin extra-cellular concentration was 5 µM and induced a Ca^2+^ fluorescence median signal of 1.8, (F/F_0_)-1.

### Cytotoxicity assay

Rat primary neuronal cells and HEK293T cells: Cortical tissue was dissected from a Sprague Dawley rat embryo brain obtained at embryonic day 18, and the dissociated cells were seeded onto the 60 inner wells of three poly-D-lysine-coated (10 µg/mL) 96-well plates, at 20,000 cells/cm^2^, in 100 µl of modified Neurobasal medium (Invitrogen, UK) per well. The plates were left undisturbed in a cell culture incubator at 37 °C and 5% CO_2_ for 7 days before peptide application, half of the culture medium being replaced every 2 days. HEK293T cells were cultured in a 96-well plate for 24 h at 37 °C and 5% CO_2_ in DMEM medium (Gibco) containing 10% Foetal Bovine Serum (Gibco). HEK293T cells were cultivated in a 96-well plate for 24 h.

Aβ and p3 peptide application: Aβ and p3 oligomers were obtained at the end of the lag-phase, with ThT in adjacent wells, while fibrils were obtained at the end of fibril assembly at equilibrium. The p3 and Aβ preparations were applied at a final concentration of 10 µM by removing half the volume of medium in each well and replacing it with an equivalent volume of p3_42;_ p3_40;_ Aβ_42_ or Aβ_40_ peptide preparations. HEPES buffer (20 mM, pH 7.4) was added to the wells that served as the controls. The cells were left undisturbed for 24 h post-treatment before their viability was assessed.

Cell viability assay: Cell viability was measured by the MTT assay. The MTT reagent (10 μL; 5 mg mL^-1^ purchased from Merck) was added to each well and left to incubate for 4 h at 37 °C. Isopropanol with 0.04 M HCl (10 μL) was added to each well. The concentration of MTT formazan produced by live cells was measured using the absorbance at 570 nm in a microplate reader (FLUOstar Omega, BMG LABTECH) within 1 h. Cell viability was presented relative to that of cells with an amount of buffer added to the cells equal to that used for peptide treatments.

### Patch clamp electrophysiology

Cell culture: HEK293 immortal cells underwent incubation in a 30 ml cell culture flask, at 37 °C, with a 5% CO_2_ environment in Dulbecco’s Modified Eagle Medium, supplemented with 10% fetal bovine serum, and penicillin-streptomycin (0.2 mg ml^-1^) mixture. Cells were subjected to division when reaching ~70–80% confluency, at intervals of ~5–7 days, employing Ca^2+^ and Mg^2+-^free phosphate-buffered saline (pH 7.2), meanwhile a portion of the cells was seeded onto 35 mm diameter easy-grip Petri dishes with the same buffer. These cells were allowed to incubate for 2-3 days prior to patch-clamp recordings.

Patch clamp recordings: All patch clamp measurements were conducted using excised membrane in an ‘inside-out’ configuration. This type of configuration facilitating the exposure of the Aβ and p3 oligomers, to the extracellular surface of plasma membrane of the HEK293 cells, while under a voltage-clamp mode. Oligomers were obtained at the end of the lag-phase of fibril assembly. Tip burnt polished micro glass pipettes (GC150TF-10, Harvard Apparatus) were backfilled with extracellular buffer containing NaCl (120 mM); CsCl (10 mM); HEPES (9 mM); KCl (2.2 mM); and MgCl_2_ (1.9 mM); CaCl_2_ (1.8 mM) and EGTA (0.1 mM) with addition of 5 μM (monomer equivalent) of Aβ or p3 oligomers at pH 7.4. Membrane patches measuring ~1–3 μm in diameter were pulled, and Aβ and p3 oligomers were permitted to diffuse towards the extracellular membrane surface within the pipette. The pipette’s resistance ranged from 2–6 MΩ when filled with the recording solution. Junction potentials manifested at the interfaces of ionic asymmetry and were duly accounted for the application of a pipette offset potential. Recordings were sampled at a frequency of 2 kHz with intervals of 500 µs, employing a lowpass 4-pole Bessel filter with a frequency cutoff of 1 kHz. The holding potential was established at -60 mV.

Channel-induced transmembrane currents were recorded by voltage clamping for 30–45 min. Employing the Axopatch 200B amplifier (Axon Instruments), a protocol ranging from -80 to 0 to 80 mV was implemented using pCLAMP 11 software. The processing of all recordings was conducted using Clampfit software, with the application of a lowpass boxcar filter.

### Reporting summary

Further information on research design is available in the Nature Portfolio Reporting Summary linked to this article.

## Supplementary information

Supplementary Information

Reporting Summary

Transparent Peer Review file

## Data Availability

All data supporting the results of this study can be found in the article, supplementary, and source data files. Source data are provided with this paper. Mass spectrometry data has been deposited in Figshare [10.6084/m9.figshare.28343099].
