# Supplementary material for: The p3 peptides (Aβ17-40/42) rapidly form amyloid fibrils that cross-seed with full-length Aβ
Source: Nat Commun. 2025 Feb 27;16:2040. doi: 10.1038/s41467-025-57341-4 (PMC11868391; doi:10.1038/s41467-025-57341-4)
Supplement: Supplementary file 1 — Supplementary Information [file 41467_2025_57341_MOESM1_ESM.pdf]

Supplementary Information

The p3 peptides (Aβ$_{17-40/42}$) rapidly form amyloid fibrils that cross-seed with full-length Aβ

Yao Tian,[1] Andrea P. Torres-Flores,[1] Qi Shang,[1] Hui Zhang,[1] Anum Khursheed,[1] Bogachan Tahirbegi,[1] Patrick N. Pallier,[2] and John H. Viles[1,*]

[1] Department of Biochemistry, School of Biological and Behavioural Sciences, Queen Mary University of London, London E1 4NS, UK

[2] The Blizard Institute, Centre for Neuroscience, Surgery and Trauma, Queen Mary University of London, London E1 2AT, UK

*Correspondence to: j.viles@qmul.ac.uk

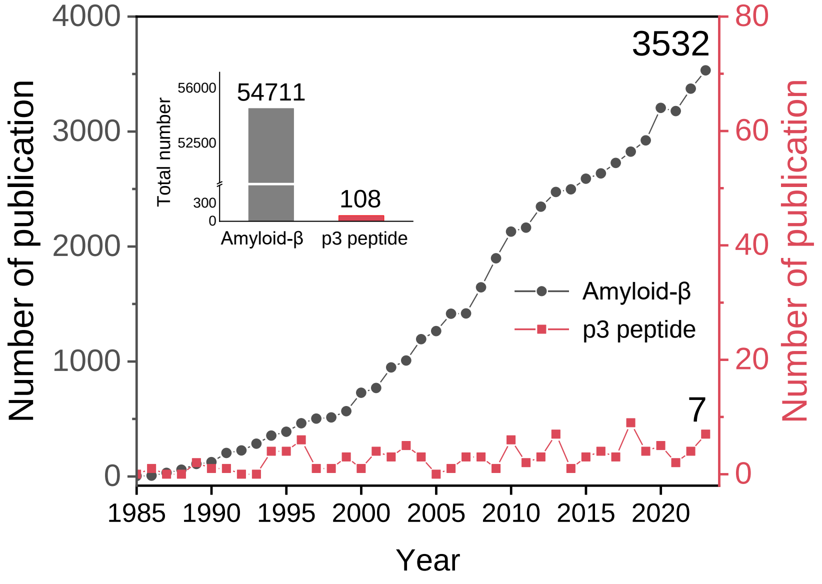

Supplementary Fig. 1 Comparison the number of papers per year published for "Amyloid-β" and "p3 peptide" since 1985. Data were searched by PubMed by using key word "Amyloid-β" and "p3 peptide" in the title. Insert: total number of papers published for "Amyloid-β" and "p3 peptide".

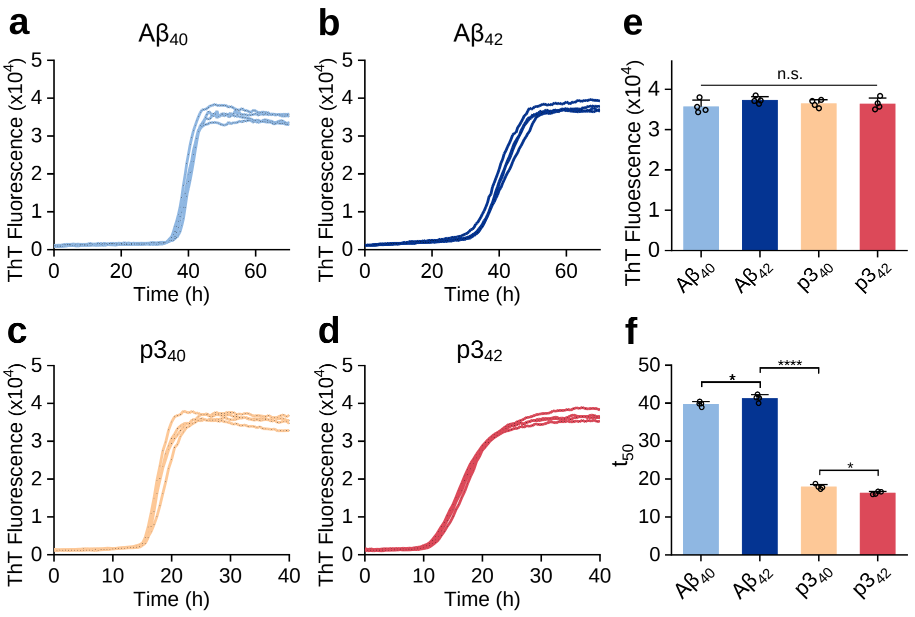

Supplementary Fig. 2 Comparison of Aβ and p3 peptides fibril kinetics curves for (a) Aβ$_{40}$, (b) Aβ$_{42}$, (c) p3$_{40}$ and (d) p3$_{42}$. e Mean maximal ThT fluorescence intensity at fibril equilibrium. **f** Comparison of t_{50} for the four peptides under the same conditions. Monomeric p3 peptides form fibrils in half the time of Aβ and very similar ThT fluorescence intensities. SEC purified monomeric preparations (10 μM) were incubated quiescently in 30 mM sodium phosphate buffer, pH 7.4. Error bars are SEM from four replicates. One-way ANOVA test, the symbols * and ****, indicate *P* values of Aβ$_{40}$-Aβ$_{42}$: $P = 0.0376$, p3$_{40}$-p3$_{42}$: $P = 0.0264$ and ****$P \leqslant 0.0001$, respectively. (This data is also shown as un-seeded controls in Supplementary Fig. 8.)

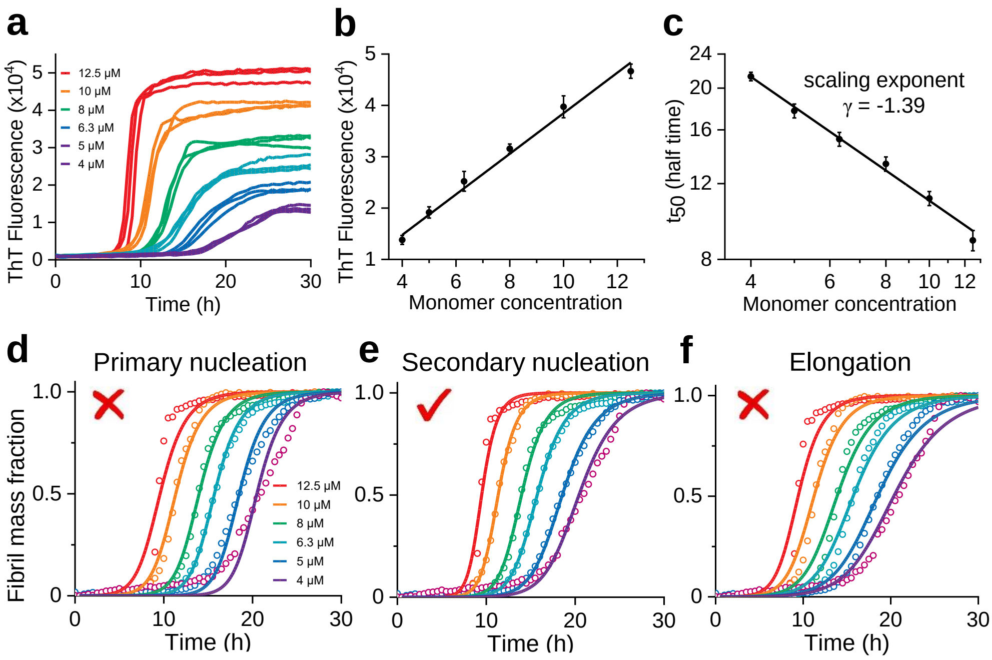

Supplementary Fig. 3 Concentration dependent aggregation of p3$_{40}$. a Kinetics profiles of p3$_{40}$ at initial monomer concentration from 4 μM (purple) to 12.5 μM (red). **b** ThT fluorescence at the plateau-phase against the p3$_{40}$ monomer concentrations. **c** Double logarithmic plot of half-time (t_{50}) versus the initial monomer concentrations, scaling exponent γ = -1.39. Error bars are SEM from three replicates. **d-f** Global fits of the kinetic traces when only primary nucleation (**d**), secondary nucleation (**e**) and fibril elongation (**f**) rate constants are altered to globally fit concentration dependent traces. Kinetic traces fit well to secondary nucleation with a low mean residual error (MRE = 0.00079), while primary nucleation and fibril elongation fit less well (MRE = 0.0023; 0.0034 respectively).

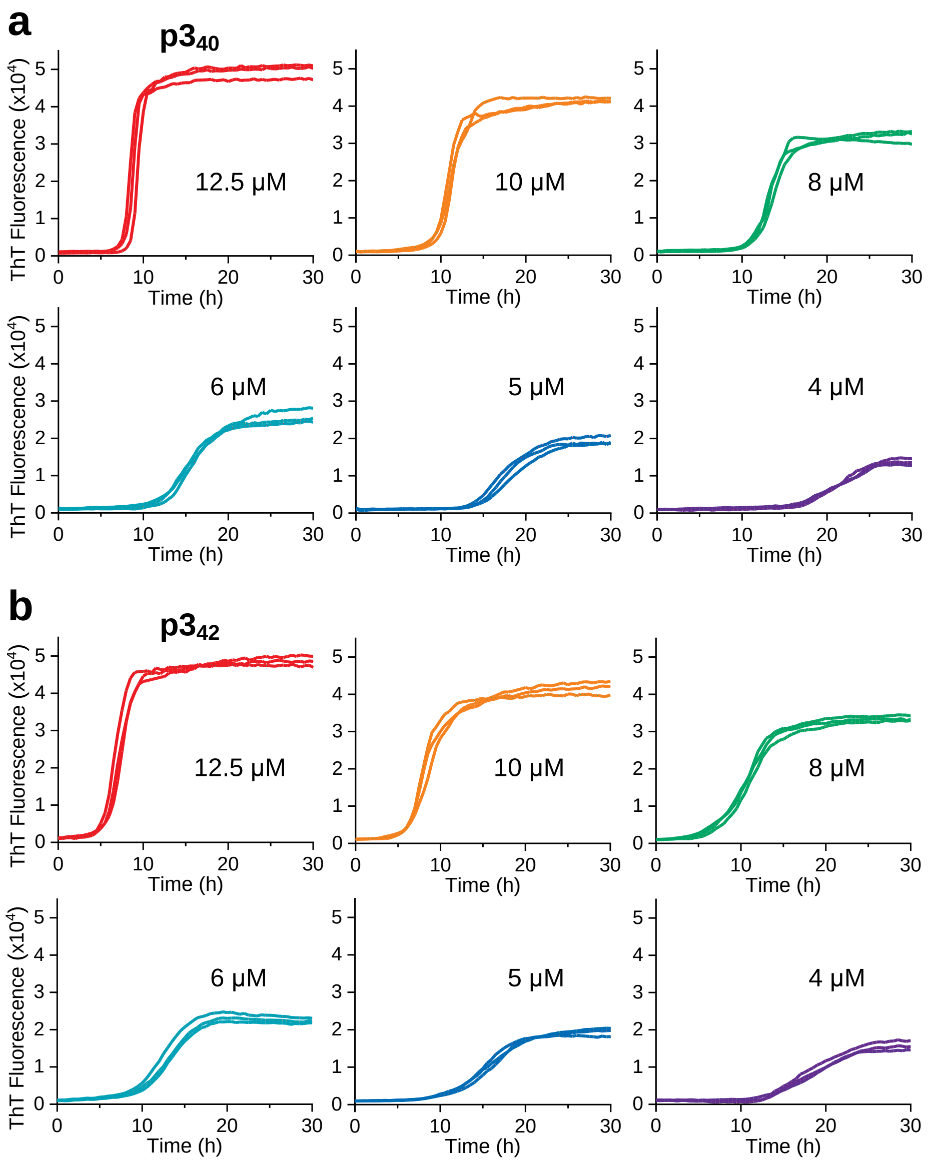

Supplementary Fig. 4 Concentration dependent aggregation of p3$_{40}$ (a) and p3$_{42}$ (b). Kinetics profiles at initial monomer concentration from 12.5 μM (red) to 4 μM (purple). N=3 traces for each condition.

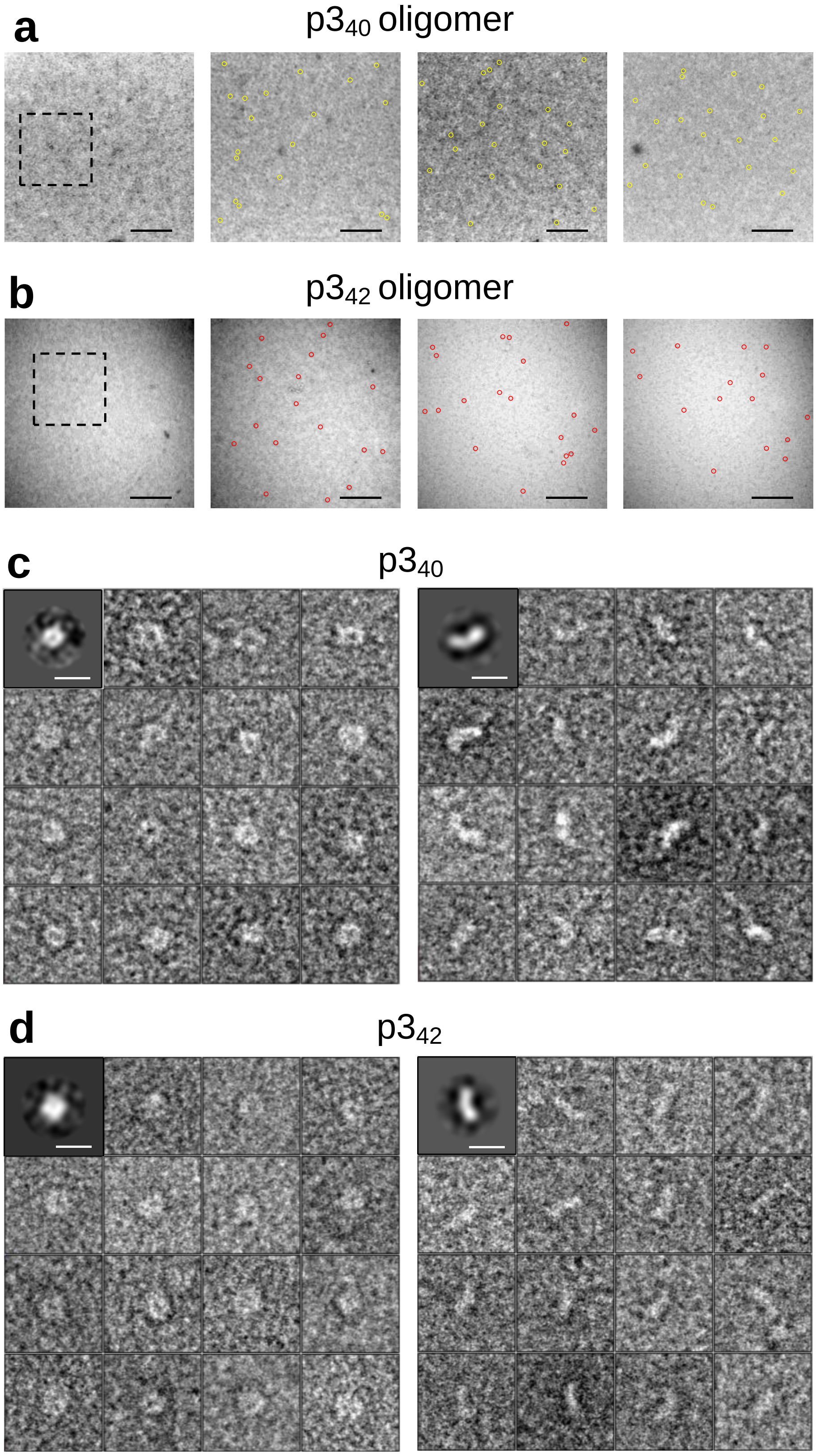

Supplementary Fig. 5 TEM images of p3$_{40}$ (a) and p3$_{42}$ (b) oligomers. The dash boxes represent images of p3 oligomers shown in Fig. 3 a, b. Scale bars: 100 nm. **c-d** Single particles from individual micrographs of p3$_{40}$ (**c**) and p3$_{42}$ (**d**) oligomers and protofibrils. Scale bars: 10 nm.

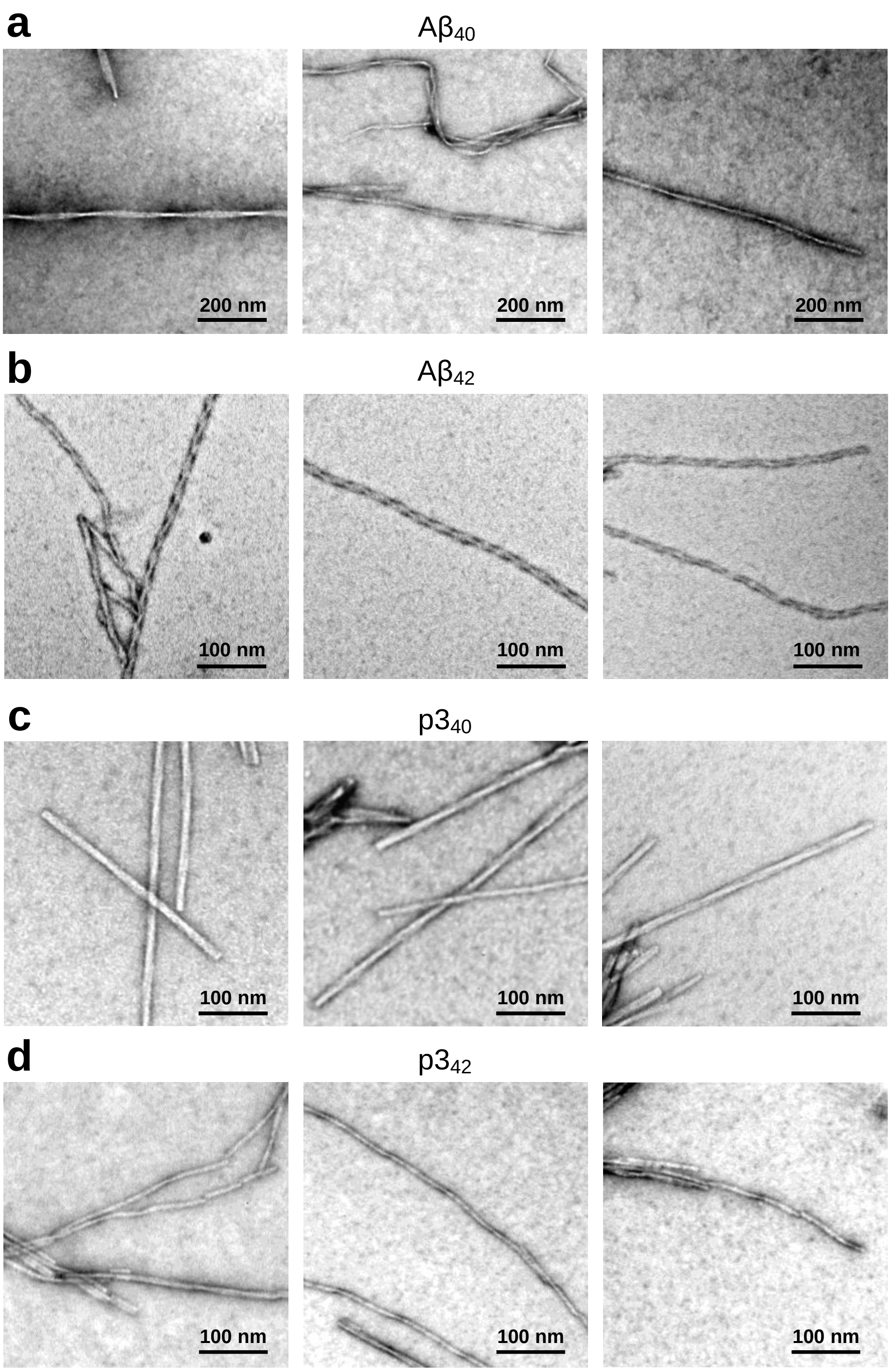

Supplementary Fig. 6 TEM fibril images of Aβ peptides. (**a**) $A\beta_{40}$, (**b**) $A\beta_{42}$, (**c**) $p3_{40}$ and (**d**) $p3_{42}$. Fibrils are negatively stained with uranyl acetate, incubated quiescently from 10 µM peptide, pH 7.4. The morphology of the Aβ peptides were reproducible and consistent on at least three independent experiments.

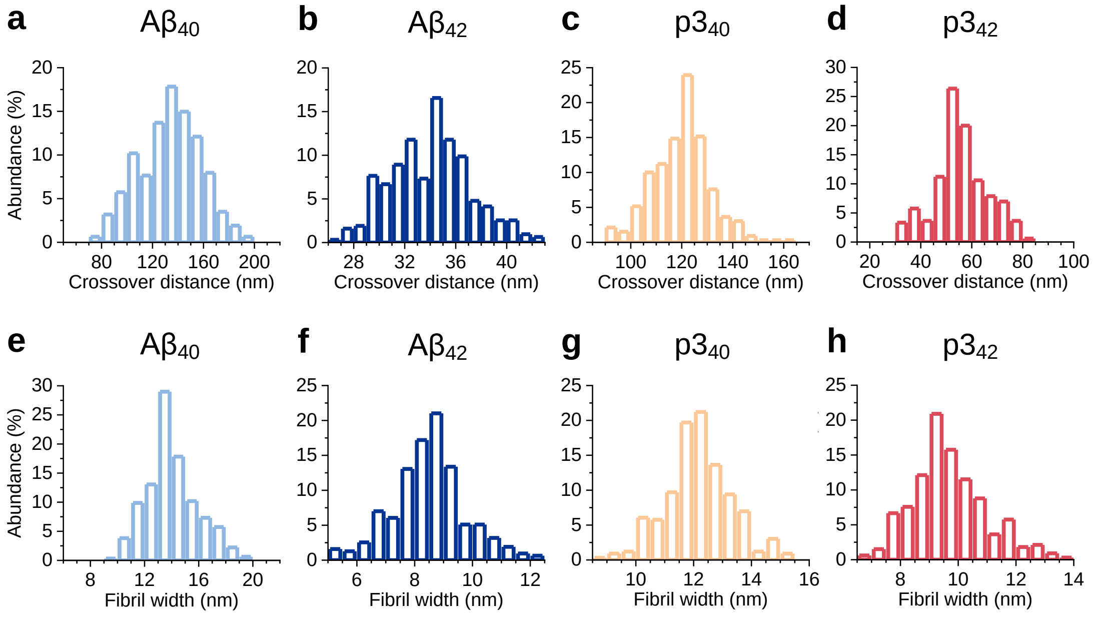

Supplementary Fig. 7 Histogram of the distribution of crossover distance (a-d) and fibril width (e-h) present in Fig. 3. Typically n>300 fibril were measured per condition.

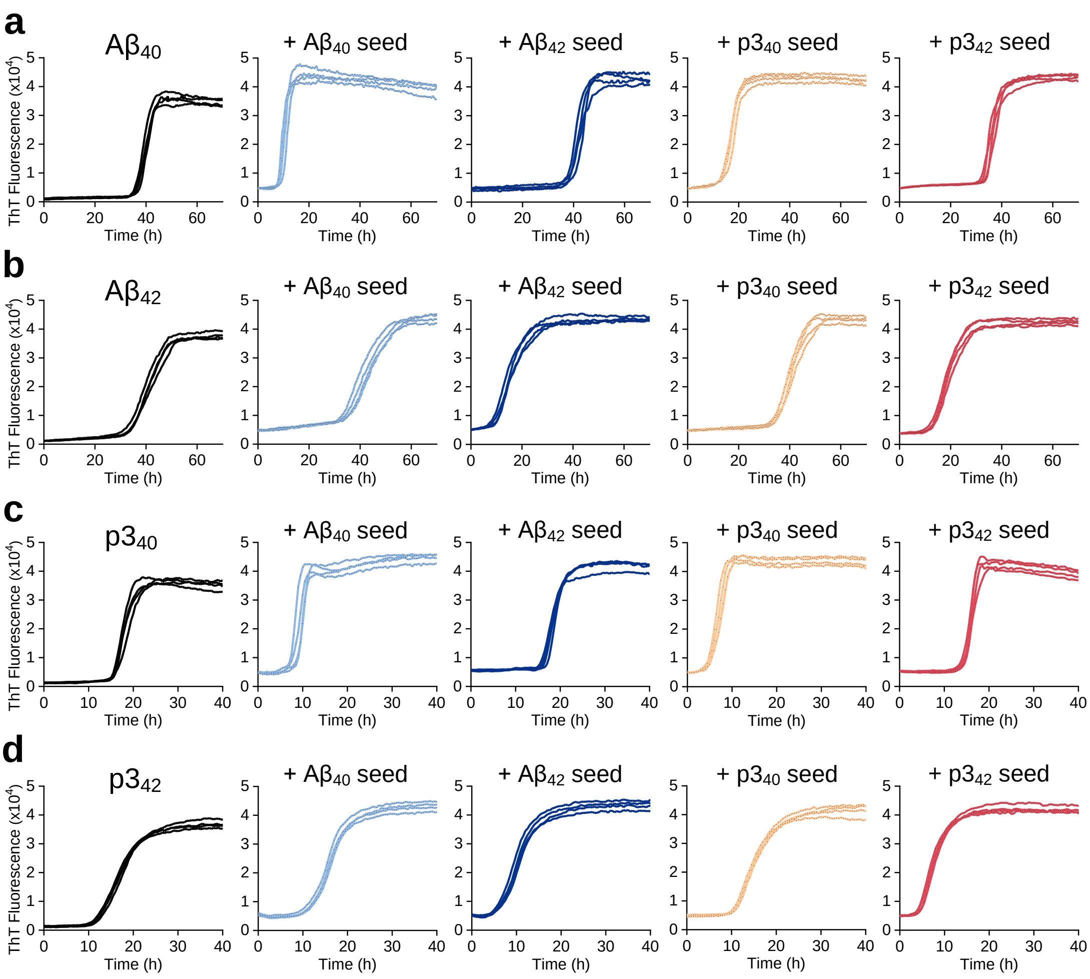

Supplementary Fig. 8 Aβ cross-seeding with p3 peptides. Fibril formation of monomeric Aβ$_{40}$ (**a**), Aβ$_{42}$ (**b**), p3$_{40}$ (**c**) and p3$_{42}$ (**d**) in presence of a range Aβ isoform fibril seeds (10 % w/w): No seed (black); Aβ$_{40}$ (pale blue); Aβ$_{42}$ (dark blue); p3$_{42}$ (orange); and p3$_{42}$ (red).

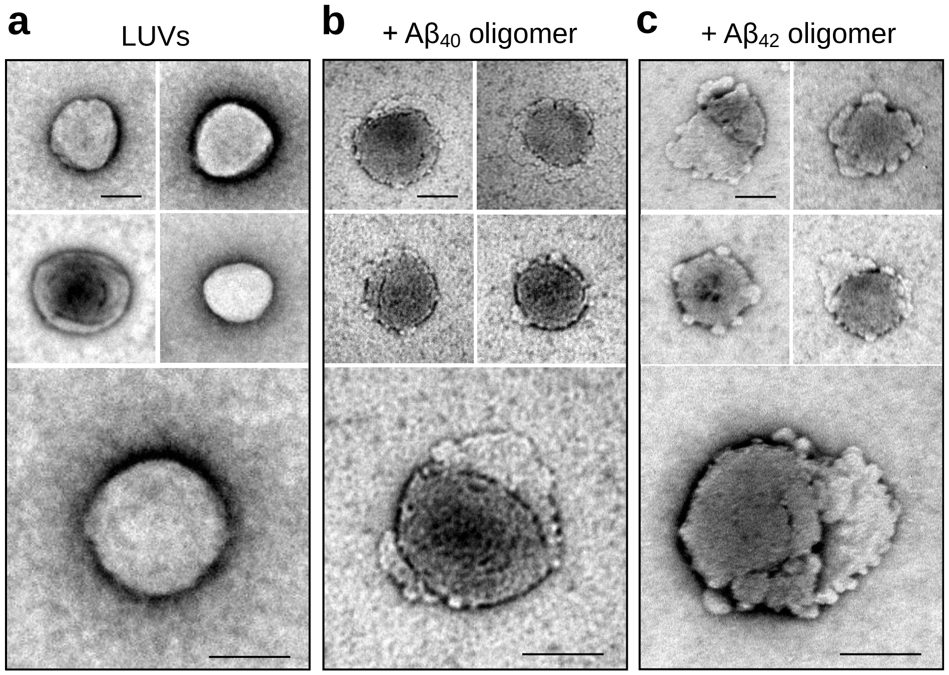

Supplementary Fig. 9 Impact of $A\beta_{40}$ and $A\beta_{42}$ oligomers on lipid vesicles. **a** Large unilamellar vesicles (LUVs) in the absence of Aβ. **b-c** LUVs incubated with $A\beta_{40}$ oligomers (**b**) and $A\beta_{42}$ oligomers (**c**). Scale bar 100 nm. Negatively stained with uranyl acetate.

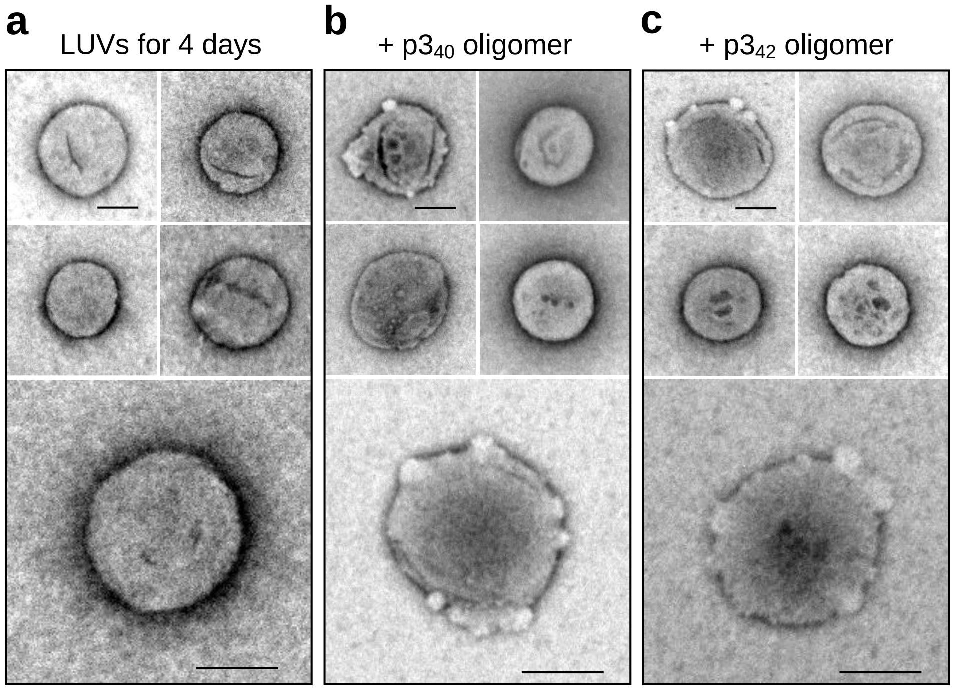

Supplementary Fig. 10 Impact of $p3_{40}$ and $p3_{42}$ oligomers on lipid vesicles. (**a**) Large unilamellar vesicles (LUVs) in the absence of p3. (**b-c**) LUVs incubated with $p3_{40}$ oligomers (**b**) and $p3_{42}$ oligomers (**c**). Scale bar 100 nm. Negatively stained with uranyl acetate.

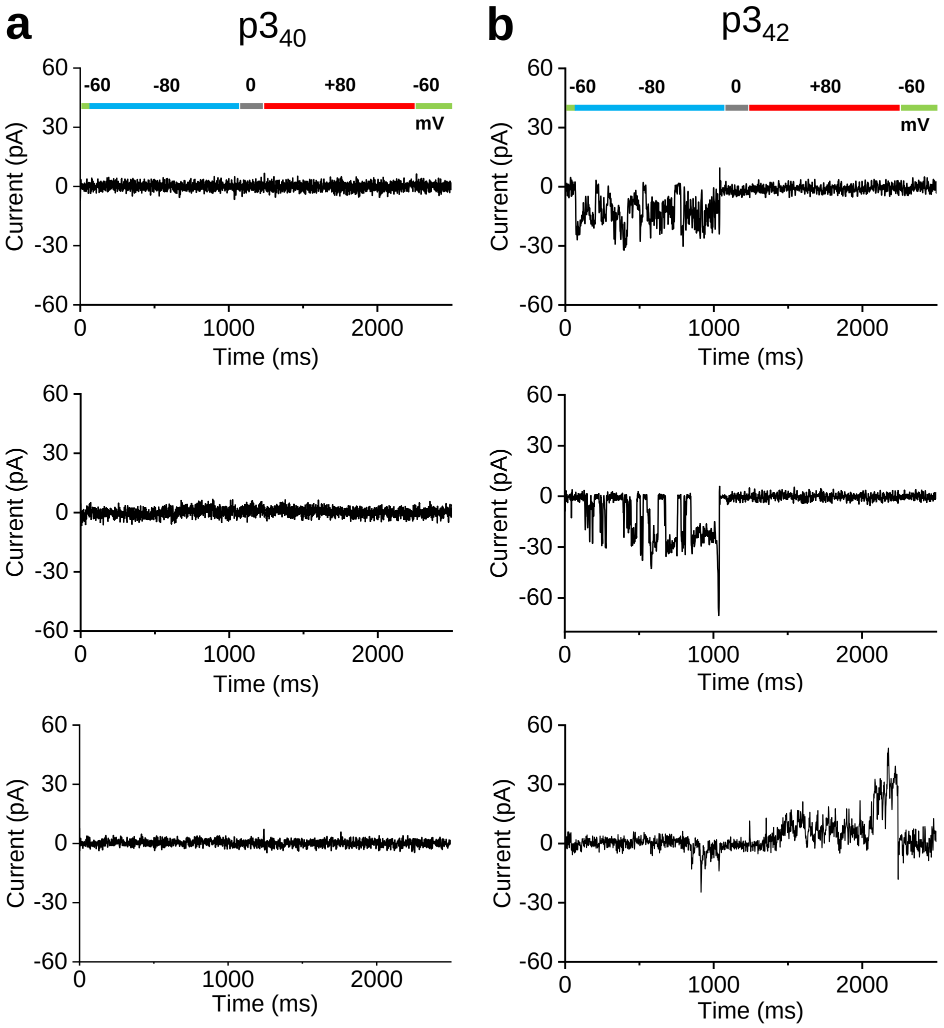

Supplementary Fig. 11 Ion channels of p3 oligomers. (**a**) p3$_{40}$ oligomers. (**b**) p3$_{42}$ oligomers. Patch-clamp current recordings, 2.5 s, across HEK293 cell membrane patches, with extra-cellular p3 oligomers (5 µM). The p3 oligomer are obtained at the end of the lag-phase during amyloid assembly. Like Aβ$_{40}$ no conductance are observed for the p3$_{40}$ peptide, while conductance of typically 200-300 pS, are observed in the p3$_{42}$ preparations.

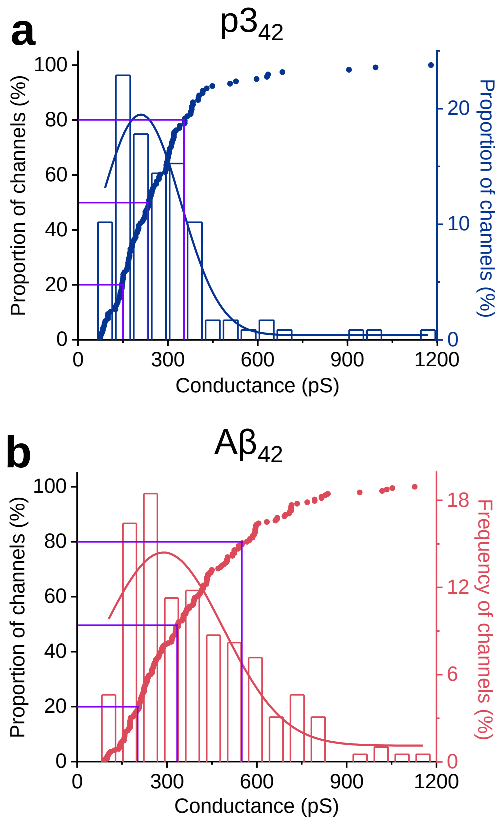

Proportion of channels (%)	Conductance (pS)
20%	150 pS
50%	200 pS
80%	355 pS

Proportion of channels (%)	Conductance (pS)
20%	200 pS
50%	340 pS
80%	575 pS

Supplementary Fig. 12 Comparison of the range of conductance for p3$_{42}$ and Aβ$_{42}$. The conductance distributions were 118 (2.5 s) measurements for p3$_{42}$ (n=5 patches) (**a**), and for Aβ$_{42}$ 195 (2.5 s) measurements (n=25 patches) (**b**). Median conductance value for p3$_{42}$ oligomers is 200 pS, whereas for Aβ$_{42}$ is 340 pS, the range of values 20%-80% maximum conductance values are also shown.

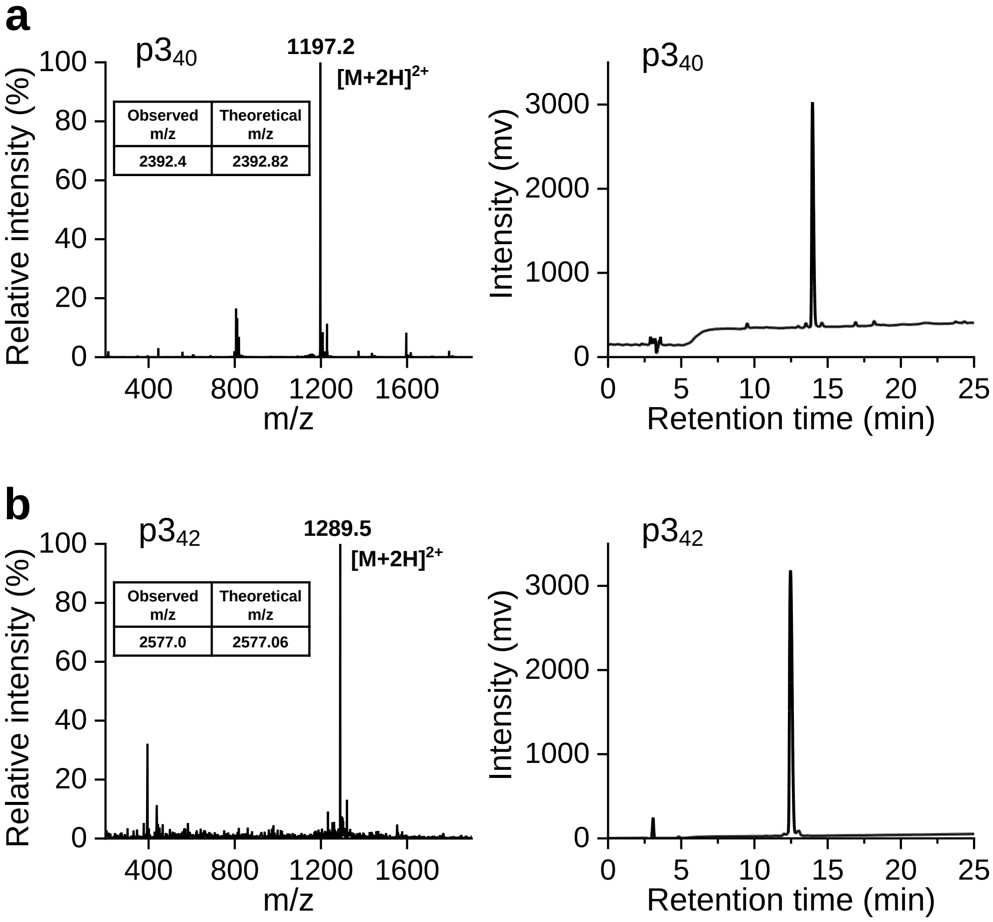

Supplementary Fig. 13 Mass spectrometry characterization and high-performance liquid chromatography (HPLC) of (a) p3$_{40}$ and (b) p3$_{42}$. The observed mass of 2392.4 is in agreement with the theoretical mass of 2392.82 for p3$_{40}$, and the observed mass of 2577.0 is in agreement with the theoretical mass of 2577.06 for p3$_{42}$.

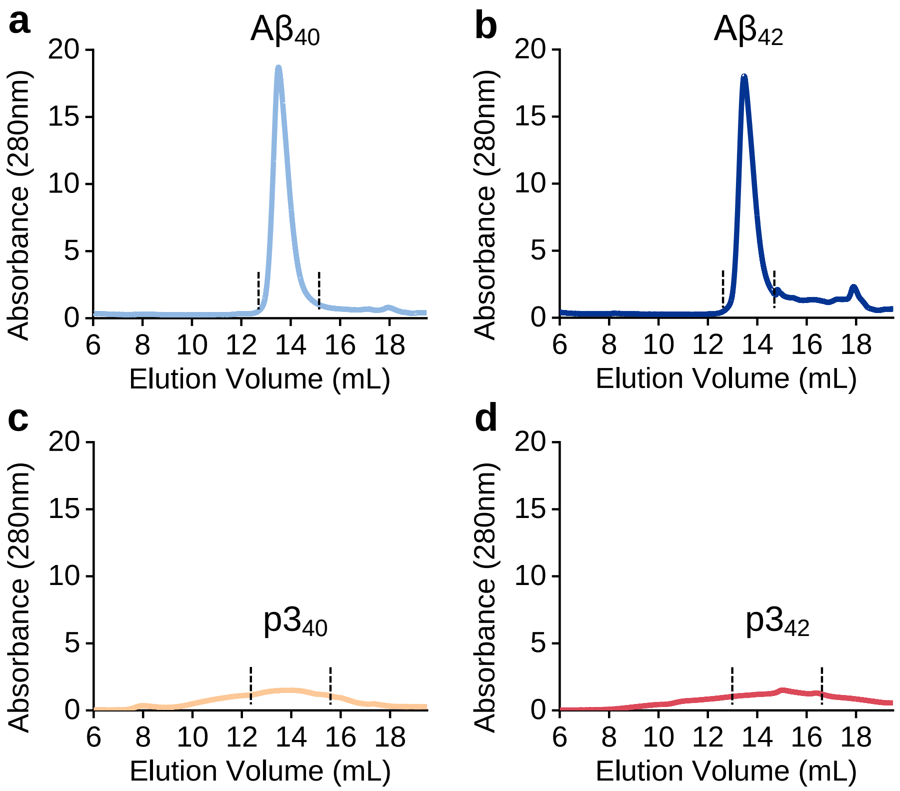

Supplementary Fig. 14 Isolation of Aβ and p3 monomer. a-b SEC elution profile (280 nm) indicates the elution of a single monomeric fraction of (**a**) $A\beta_{40}$ and (**b**) $A\beta_{42}$. **c-d** $p3_{40}$ and $p3_{42}$ sequences lack a tyrosine residue (Tyr10), so the absorbance at 280 nm is low, and monomer fraction taken form elution volume between 12-16 mL. p3 peptides concentrations were determined by the amide absorbance at 205 nm (see methods). The Aβ and p3 monomeric samples were taken directly from the SEC column elution.

Supplementary Table 1. Comparison of mean cross-over length and fibril width for $A\beta_{40}$, $A\beta_{42}$, $p3_{40}$ and $p3_{42}$ fibrils. The fibrils for each peptide have a range of twists and widths, which are represented as histograms in supplementary Fig. 7.

	mean crossover distance (nm)	mean fibril width (nm)
$A\beta_{40}$	133.8	14.0
$p3_{40}$	120.3	12.2
$A\beta_{42}$	34.1	8.5
$p3_{42}$	55.9	9.7

Supplementary Table 2. Tabulated t_{50} for seeding conditions relative to non-seeded monomer, taken from the data shown in Fig. 4.

Monomer \ Seeds	No Seed	$A\beta_{40}$	$A\beta_{42}$	$p3_{40}$	$p3_{42}$
$A\beta_{40}$	39.9 h	10.4 h	42.5 h	17.4 h	36.3 h
$A\beta_{42}$	40.5 h	41.9 h	15.1 h	40.3 h	18.7 h
$p3_{40}$	18.0 h	9.4 h	18.5 h	6.9 h	16.0 h
$p3_{42}$	16.8 h	16.0 h	10.1 h	14.8 h	7.3 h

Supplementary Table 3. Analysis of liposomes (LUVs) disrupted by Aβ_{40}, Aβ_{40}, p3$_{40}$ and p3$_{42}$ oligomers for negatively stained samples.

Liposome	Independent preparation	Number of vesicles inspected	% of vesicles decorated
Buffer	a	100	0%
Buffer	b	103	0%
Buffer	c	101	0%
Total / Mean		***304***	***0%***
Aβ_{40}	a	98	78%
Aβ_{40}	b	96	84%
Aβ_{40}	c	104	81%
Total / Mean		***298***	***81%***
Aβ_{42}	a	102	82%
Aβ_{42}	b	101	83%
Aβ_{42}	c	98	85%
Total / Mean		***301***	***83%***
p3$_{40}$	a	102	32%
p3$_{40}$	b	102	30%
p3$_{40}$	c	104	33%
Total / Mean		***308***	***32%***
p3$_{42}$	a	104	34%
p3$_{42}$	b	101	36%
p3$_{42}$	c	105	34%
Total / Mean		***310***	***35%***

Supplementary Table 4. Membrane patches recorded with ion channel for Aβ_{40}, Aβ_{42}, p3$_{40}$ and p3$_{42}$ oligomers**.**

Peptide	Recorded Patches	Patches with channels	% of patches with channels
Aβ_{40}	50	0	0 %
Aβ_{42}	72	25	35 %
p3$_{40}$	30	0	0 %
p3$_{42}$	32	5	16 %
